# Hierarchical Models in the Brain

**DOI:** 10.1371/journal.pcbi.1000211

**Published:** 2008-11-07

**Authors:** Karl Friston

**Affiliations:** The Wellcome Trust Centre of Neuroimaging, University College London, London, United Kingdom; Indiana University, United States of America

## Abstract

This paper describes a general model that subsumes many parametric models for continuous data. The model comprises hidden layers of state-space or dynamic causal models, arranged so that the output of one provides input to another. The ensuing hierarchy furnishes a model for many types of data, of arbitrary complexity. Special cases range from the general linear model for static data to generalised convolution models, with system noise, for nonlinear time-series analysis. Crucially, all of these models can be inverted using exactly the same scheme, namely, dynamic expectation maximization. This means that a single model and optimisation scheme can be used to invert a wide range of models. We present the model and a brief review of its inversion to disclose the relationships among, apparently, diverse generative models of empirical data. We then show that this inversion can be formulated as a simple neural network and may provide a useful metaphor for inference and learning in the brain.

## Author Summary

Models are essential to make sense of scientific data, but they may also play a central role in how we assimilate sensory information. In this paper, we introduce a general model that generates or predicts diverse sorts of data. As such, it subsumes many common models used in data analysis and statistical testing. We show that this model can be fitted to data using a single and generic procedure, which means we can place a large array of data analysis procedures within the same unifying framework. Critically, we then show that the brain has, in principle, the machinery to implement this scheme. This suggests that the brain has the capacity to analyse sensory input using the most sophisticated algorithms currently employed by scientists and possibly models that are even more elaborate. The implications of this work are that we can understand the structure and function of the brain as an inference machine. Furthermore, we can ascribe various aspects of brain anatomy and physiology to specific computational quantities, which may help understand both normal brain function and how aberrant inferences result from pathological processes associated with psychiatric disorders.

## Introduction

This paper describes hierarchical dynamic models (HDMs) and reviews a generic variational scheme for their inversion. We then show that the brain has evolved the necessary anatomical and physiological equipment to implement this inversion, given sensory data. These models are general in the sense that they subsume simpler variants, such as those used in independent component analysis, through to generalised nonlinear convolution models. The generality of HDMs renders the inversion scheme a useful framework that covers procedures ranging from variance component estimation, in classical linear observation models, to blind deconvolution, using exactly the same formalism and operational equations. Critically, the nature of the inversion lends itself to a relatively simple neural network implementation that shares many formal similarities with real cortical hierarchies in the brain.

Recently, we introduced a variational scheme for model inversion (i.e., inference on models and their parameters given data) that considers hidden states in generalised coordinates of motion. This enabled us to derive estimation procedures that go beyond conventional approaches to time-series analysis, like Kalman or particle filtering. We have described two versions; variational filtering [1] and dynamic expectation maximisation (**DEM**; [2]) that use free and fixed-form approximations to the posterior or conditional density respectively. In these papers, we used hierarchical dynamic models to illustrate how the schemes worked in practice. In this paper, we focus on the model *per se* and the relationships among its special cases. We will use **DEM** to show how their inversion relates to conventional treatments of these special cases.

A key aspect of **DEM** is that it was developed with neuronal implementation in mind. This constraint can be viewed as formulating a neuronally inspired estimation and inference framework or conversely, as providing heuristics that may inform our understanding of neuronal processing. The basic ideas have already been described, in the context of static models, in a series of papers [3]–[5] that entertain the notion that the brain may use empirical Bayes for inference about its sensory input, given the hierarchical organisation of cortical systems. In this paper, we generalise this idea to cover hierarchical dynamical systems and consider how neural networks could be configured to invert HDMs and deconvolve sensory causes from sensory input.

This paper comprises five sections. In the first, we introduce hierarchical dynamic models. These cover many observation or generative models encountered in the estimation and inference literature. An important aspect of these models is their formulation in generalised coordinates of motion; this lends them a hierarchal form in both structure and dynamics. These hierarchies induce empirical priors that provide structural and dynamic constraints, which can be exploited during inversion. In the second and third sections, we consider model inversion in general terms and then specifically, using dynamic expectation maximisation (**DEM**). This reprises the material in Friston et al. [2] with a special focus on HDMs. **DEM** is effectively a variational or ensemble learning scheme that optimises the conditional density on model states (**D**-step), parameters (**E**-step) and hyperparameters (**M**-step). It can also be regarded as a generalisation of expectation maximisation (**EM**), which entails the introduction of a deconvolution or **D**-step to estimate time-dependent states. In the fourth section, we review a series of HDMs that correspond to established models used for estimation, system identification and learning. Their inversion is illustrated with worked-examples using **DEM**. In the final section, we revisit the **DEM** steps and show how they can be formulated as a simple gradient ascent using neural networks and consider how evoked brain responses might be understood in terms of inference under hierarchical dynamic models of sensory input.

### Notation

To simplify notation we will use *f_x_*: = *f_x_*(*x*) = ∂*_x_f* = ∂*f*/∂*x* to denote the partial derivative of the function, *f*, with respect to the variable *x*. We also use *x* ˙ = ∂*_t_x* for temporal derivatives. Furthermore, we will be dealing with variables in generalised coordinates of motion, which will be denoted by a tilde; *x̃*: = [*x*,*x*′,*x*″,…]*^T^* = [*x* ^[0]^,*x* ^[1]^,*x* ^[2]^,…]*^T^*, where *x* ^[*i*]^ denotes *i*th order motion. A point in generalised coordinates can be regarded as encoding the instantaneous trajectory of a variable, in the sense it prescribes its location, velocity, acceleration etc.

## Materials and Methods

### Hierarchical Dynamic Models

In this section, we cover hierarchal models for dynamic systems. We start with the basic model and how generalised motion furnishes empirical priors on the dynamics of the model's hidden states. We then consider hierarchical forms and see how these induce empirical priors in a structural sense. We will try to relate these perspectives to established treatments of empirical priors in static and state-space models.

#### Hierarchical dynamic causal models

Dynamic causal models are probabilistic generative models *p*(*y*,*ϑ*) based on state-space models. As such, they entail the likelihood, *p*(*y*|*ϑ*) of getting some data, *y*, given some parameters *ϑ* = {*x*,*v*,*θ*,*λ*} and priors on those parameters, *p*(*ϑ*). We will see that the parameters subsume different quantities, some of which change with time and some which do not. These models are causal in a control-theory sense because they are state-space models, formulated in continuous time.

#### State-pace models in generalised coordinates

A dynamic input-state-output model can be written as
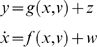
(1)The continuous nonlinear functions *f* and *g* of the states are parameterised by *θ*. The states *v*(*t*) can be deterministic, stochastic, or both. They are variously referred to as inputs, sources or causes. The states *x*(*t*) meditate the influence of the input on the output and endow the system with memory. They are often referred to as hidden states because they are seldom observed directly. We assume the stochastic terms (i.e., observation noise) *z*(*t*) are analytic, such that the covariance of *z̃* = [*z*,*z*′,*z*″,…]*^T^* is well defined; similarly for the system or state noise, *w*(*t*), which represents random fluctuations on the motion of the hidden states. Under local linearity assumptions (i.e., ignoring high-order derivatives of the generative model functions), the generalised output or response *ỹ* = [*y*,*y*′,*y*″,…]*^T^* obtains from recursive differentiation with respect to time using the chain rule
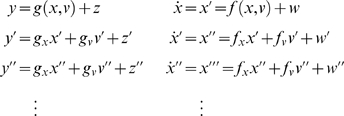
(2)Note that the derivatives are evaluated at each point in time and the linear approximation is local to the current state. The first (observer) equation show that the generalised states *u* = [*ṽ*,*x̃*,]*^T^* are needed to generate a generalised response that encodes a path or trajectory. The second (state) equations enforce a coupling between neighbouring orders of motion of the hidden states and confer memory on the system.

At this point, readers familiar with standard state-space models may be wondering where all the extra equations in Equation 2 come from and, in particular, what the generalised motions; *w*′, *w*″, … represent. These terms always exist but are ignored in standard treatments based on the theory of Markovian processes [6]. This is because standard Markovian (c.f., Wiener) processes have generalised motion that has infinite variance and are infinitely ‘jagged’ or rough. This means *w*′, *w*″, … and *x*″, *x*‴, … have no precision (inverse variance) and can be ignored with impunity. It is important to realise that this approximation is not appropriate for real or actual fluctuations, as noted at the inception of the standard theory; “a certain care must be taken in replacing an actual process by Markov process, since Markov processes have many special features, and, in particular, differ from the processes encountered in radio engineering by their lack of smoothness… any random process actually encountered in radio engineering is analytic, and all its derivative are finite with probability one” ([6], pp 122–124). So why have standard state-space models, and their attending inversion schemes like Kalman filtering, dominated the literature over the past half-century? Partly because it is convenient to ignore generalised motion and partly because they furnish reasonable approximations to fluctuations over time-scales that exceed the correlation time of the random processes: “Thus the results obtained by applying the techniques of Markov process theory are valuable only to the extent to which they characterise just these ‘large-scale’ fluctuations” ([6], p 123). However, standard models fail at short time-scales. This is especially relevant in this paper because the brain has to model continuous sensory signals on a fast time-scale.

Having said this, it is possible to convert the generalised state-space model in Equation 2 into a standard form by expressing the components of generalised motion in terms of a standard [uncorrelated] Markovian process, *ς*(*t*):
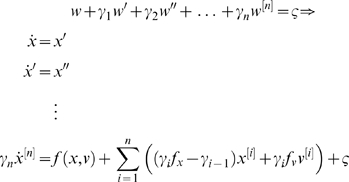
(3)The first line encodes the autocorrelation function or spectral density of the fluctuations *w*(*t*) in term of smoothness parameters, *γ*_1_,…*γ_n_*, where *n* is the order of generalised motion. These parameters can be regarded as the coefficients of a polynomial expansion *P_n_*(∂*_t_*)*w* = *ς*(*t*) (see [6], Equation 4.288 and below). The second line obtains by substituting Equation 2 into the first and prescribes a standard state-space model, whose states cover generalised motion; *x* ^[0]^,…,*x* ^[*n*]^. When *n* = 0 we recover the state equation in Equation 1, namely, *x* ˙ = *f*(*x*,*v*)+*ς*. This corresponds to the standard Markovian approximation because the random fluctuations are uncorrelated and *w* = *ς*; from Equation 3. When *n* = 1⇒*w*+*γ*_1_ *w*′ = *ς*, the fluctuations *w*(*t*) correspond to an exponentially correlated process, with a decay time of *γ*_1_ ([6], p 121). However, generally *n* = ∞: “Therefore we cannot describe an actual process within the framework of Markov process theory, and the more accurately we wish to approximate such a process by a Markov process, the more components the latter must have.” ([6], p 165). See also [7] (pp 122–125) for a related treatment.

If there is a formal equivalence between standard and generalised state-space models, why not use the standard formulation, with a suitably high-order approximation? The answer is that we do not need to; by retaining an explicit formulation in generalised coordinates we can devise a simple inversion scheme (Equation 23) that outperforms standard Markovian techniques like Kalman filtering. This simplicity is important because we want to understand how the brain inverts dynamic models. This requires a relatively simple neuronal implementation that could have emerged through natural selection. From now on, we will reserve ‘state-space models’ (SSM) for standard *n* = 0 models that discount generalised motion and, implicitly, serial correlations among the random terms. This means we can treat SSMs as special cases of generalised state-space models, in which the precision of generalised motion on the states noise is zero.

#### Probabilistic dynamic models

Given the form of generalised state-space models we now consider what they entail as probabilistic models of observed signals. We can write Equation 2 compactly as
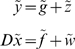
(4)Where the predicted response *g̃* = [*g*,*g*′,*g*″,…]*^T^* and motion *f̃* = [*f*,*f*′,*f*″,…]*^T^* in the absence of random fluctuations are
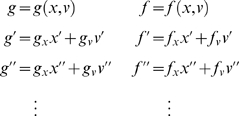
and *D* is a block-matrix derivative operator, whose first leading-diagonal contains identity matrices. This operator simply shifts the vectors of generalised motion so *x* ^[*i*]^ that is replaced by *x* ^[*i*+1]^.

Gaussian assumptions about the fluctuations 
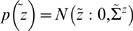
 provide the likelihood, *p*(*ỹ*|*x̃*,*ṽ*). Similarly, Gaussian assumptions about state-noise 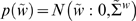 furnish empirical priors, *p*(*x̃*|*ṽ*) in terms of predicted motion
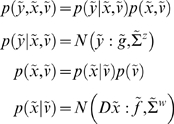
(5)We will assume Gaussian priors 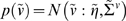 on the generalised causes, with mean 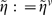 and covariance 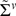. The density on the hidden states *p*(*x̃*|*ṽ*) is part of the prior on quantities needed to evaluate the likelihood of the response or output. This prior means that low-order motion constrains high-order motion (and *vice versa*). These constraints are discounted in standard state-space models because the precision on the generalised motion of a standard Markovian process is zero. This means the only constraint is mediated by the prior *p*(*x* ˙|*x*,*v*). However, it is clear from Equation 5 that high-order terms contribute. In this work, we exploit these constraints by adopting more plausible models of noise, which are encoded by their covariances 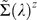 and 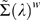 (or precisions 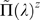 and 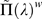). These are functions of unknown hyperparameters, *λ* which control the amplitude and smoothness of the random fluctuations.

**Figure 1:**
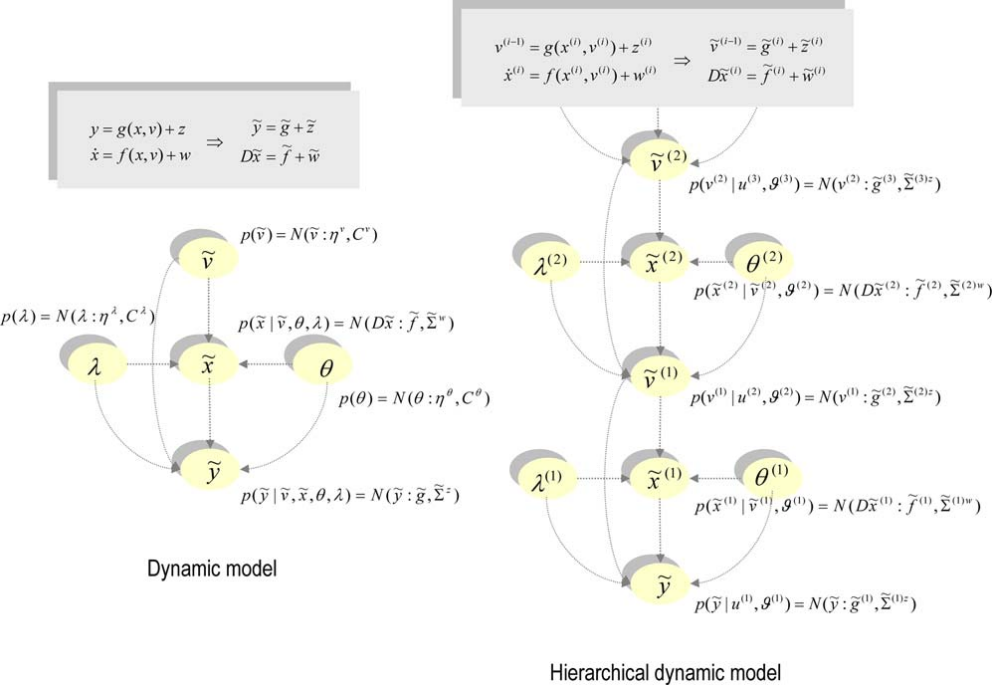
Conditional dependencies of dynamic (right) and hierarchical (left) models, shown as directed Bayesian graphs. The nodes of these graphs correspond to quantities in the model and the responses they generate. The arrows or edges indicate conditional dependencies between these quantities. The form of the models is provided, both in terms of their state-space equations (above) and in terms of the prior and conditional probabilities (below). The hierarchal structure of these models induces empirical priors; dynamical priors are mediated by the equations of generalised motion and structural priors by the hierarchical form, under which states in higher levels provide constraints on the level below.

Figure 1 (left) shows the directed graph depicting the conditional dependencies implied by this model. Next, we consider hierarchal models that provide another form of hierarchical constraint. It is useful to note that hierarchical models are special cases of Equation 1, in the sense that they are formed by introducing conditional independencies (i.e., removing edges in Bayesian dependency graphs).

#### Hierarchical forms

HDMs have the following form, which generalises the (*m* = 1) model above
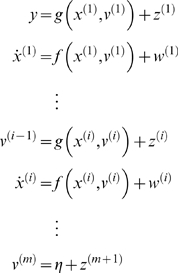
(6)Again, *f* ^(*i*)^ : = *f*(*x* ^(*i*)^,*v* ^(*i*)^) and *g* ^(*i*)^ : = *g*(*x* ^(*i*)^,*v* ^(*i*)^) are continuous nonlinear functions of the states. The processes *z* ^(*i*)^ and *w* ^(*i*)^ are conditionally independent fluctuations that enter each level of the hierarchy. These play the role of observation error or noise at the first level and induce random fluctuations in the states at higher levels. The causes *v* = [*v* ^(1)^,…,*v* ^(*m*)^]*^T^* link levels, whereas the hidden states *x* = [*x* ^(1)^,…,*x* ^(*m*)^]*^T^* link dynamics over time. The corresponding directed graphical model is shown in Figure 1 (right). In hierarchical form, the output of one level acts as an input to the next. When the state-equations are linear, the hierarchy performs successive convolutions of the highest level input, with random fluctuations entering at each level. However, inputs from higher levels can also enter nonlinearly into the state equations and can be regarded as changing its control parameters to produce quite complicated generalised convolutions with ‘deep’ (i.e., hierarchical) structure.

The conditional independence of the fluctuations at different hierarchical levels means that the HDM has a Markov property over levels, which simplifies attending inference schemes. See [8] for a discussion of approximate Bayesian inference in conditionally independent hierarchical models of static data. Consider the empirical prior implied by Equation 6
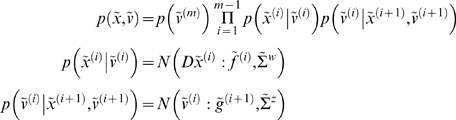
(7)where the full prior 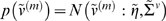 is now restricted to the last level. Equation 7 is similar in form to the prior in Equation 5 but now factorises over levels; where higher causes place empirical priors on the dynamics of the level below. The factorisation in Equation 7 is important because one can appeal to empirical Bayes to interpret the conditional dependences. In empirical Bayes [9], factorisations of the likelihood create empirical priors that share properties of both the likelihood and priors. For example, the prediction *g̃* ^(*i*)^ = *g̃*(*x̃* ^(*i*)^,*ṽ* ^(*i*)^) plays the role of a prior expectation on *ṽ* ^(*i*−1)^, yet it has to be estimated in terms of *x̃* ^(*i*)^,*ṽ* ^(*i*)^. In short, a hierarchical form endows models with the ability to construct their own priors. These formal or structural priors are central to many inference and estimation procedures, ranging from mixed-effects analyses in classical covariance component analysis to automatic relevance determination in machine learning. The hierarchical form and generalised motion in HDMs furnishes them with both structural and dynamic empirical priors respectively.

#### The precisions and temporal smoothness

In generalised coordinates, the precision, 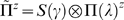 is the Kronecker tensor product of a temporal precision matrix, *S*(*γ*) and the precision over random fluctuations, which has a block diagonal form in hierarchical models; similarly for 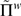. The temporal precision encodes temporal dependencies among the random fluctuations and can be expressed as a function of their autocorrelations
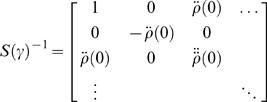
(8)Here 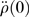 is the second derivative of the autocorrelation function evaluated at zero. This is a ubiquitous measure of roughness in the theory of stochastic processes [10]. Note that when the random fluctuations are uncorrelated, the curvature (and higher derivatives) of the autocorrelation are infinite. In this instance, the precision of high-order motion falls to zero. This is the limiting case assumed by state-space models; it corresponds to the assumption that incremental fluctuations are independent (c.f., a Wiener process or random walk). Although, this is a convenient assumption that is exploited in conventional Bayesian filtering schemes and appropriate for physical systems with Brownian processes, it is less plausible for biological and other systems, where random fluctuations are themselves generated by dynamical systems ([6], p 81).

**Figure 2:**
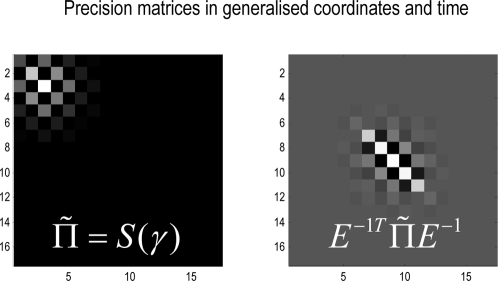
Image representations of the precision matrices encoding temporal dependencies among the generalised motion of random fluctuations. The precision in generalised coordinates (left) and over discrete samples in time (right) are shown for a roughness of *γ* = 4 and seventeen observations (with an order of *n* = 16). This corresponds to an autocorrelation function whose width is half a time bin. With this degree of temporal correlation only a few (i.e., five or six) discrete local observations are specified with any precision.

*S*(*γ*) can be evaluated for any analytic autocorrelation function. For convenience, we assume that the temporal correlations have the same Gaussian form. This gives
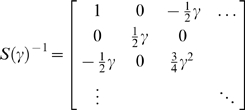
(9)Here, *γ* is the precision parameter of a Gaussian autocorrelation function. Typically, *γ*>1, which ensures the precisions of high-order motion converge quickly. This is important because it enables us to truncate the representation of an infinite number of generalised coordinates to a relatively small number; because high-order prediction errors have a vanishingly small precision. An order of *n* = 6 is sufficient in most cases [1]. A typical example is shown in Figure 2, in generalised coordinates and after projection onto the time-bins (using a Taylor expansion, whose coefficients comprise the matrix *Ẽ*). It can be seen that the precision falls quickly with order and, in this case, we can consider just six orders of motion, with no loss of precision.

When dealing with discrete time-series it is necessary to map the trajectory implicit in the generalised motion of the response onto discrete samples, [*y*(*t*_1_),…,*y*(*t_N_*)]*^T^* = *Ẽ* *ỹ*(*t*) (note that this is not necessary with continuous data such as sensory data sampled by the brain). After this projection, the precision falls quickly over time-bins (Figure 2, right). This means samples in the remote past or future do not contribute to the likelihood and the inversion of discrete time-series data can proceed using local samples around the current time bin; i.e., it can operate ‘on-line’.

#### Energy functions

We can now write down the exact form of the generative model. For dynamic models, under Gaussian assumptions about the random terms, we have a simple quadratic form (ignoring constants)
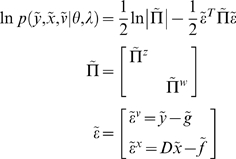
(10)The auxiliary variables 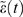 comprise prediction errors for the generalised response and motion of hidden states, where 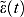 and 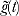 are the respective predictions, whose precision is encoded by 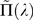. The use of prediction errors simplifies exposition and may be used in neurobiological implementations (i.e., encoded explicitly in the brain; see last section and [4]). For hierarchical models, the prediction error on the response is supplemented with prediction errors on the causes
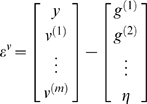
(11)Note that the data and priors enter the prediction error at the lowest and highest level respectively. At intermediate levels the prediction errors, *v* ^(*i*−1)^−*g* ^(*i*)^ mediate empirical priors on the causes. In the next section, we will use a variational inversion of the HDM, which entails message passing between hierarchical levels. These messages are the prediction errors and their influence rests on the derivatives of the prediction error with respect to the unknown states
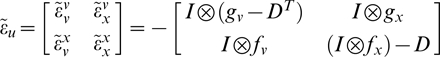
(12)This form highlights the role of causes in linking successive hierarchical levels (the *D^T^* matrix) and the role of hidden states in linking successive temporal derivatives (the *D* matrix). The *D^T^* in the upper-left block reflects the fact that that the prediction error on the causes depends on causes at that level and the lower level being predicted; *ε* ^(*i*)*v*^ = *v* ^(*i*−1)^−*g*(*x* ^(*i*)^,*v* ^(*i*)^). The *D* in the lower-right block plays a homologous role, in that the prediction error on the motion of hidden states depends on motion at that order and the higher order; *ε* ^[*i*]*x*^ = *x* ^[*i*+1]^−*f*(*x* ^[*i*]^,*v* ^[*i*]^).

These constraints on the structural and dynamic form of the system are specified by the functions *g* = [*g* ^(1)^,…,*g* ^(*m*)^]*^T^* and *f* = [*f* ^(1)^,…,*f* ^(*m*)^]*^T^*, respectively. The partial derivatives of these functions have a block diagonal form, reflecting the model's hierarchical separability
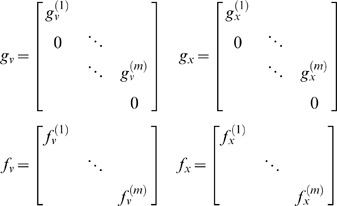
(13)Note that the partial derivatives of *g*(*x*,*v*) have an extra row to accommodate the top level. To complete model specification we need priors on the parameters and hyperparameters. We will assume these are Gaussian, where (ignoring constants)
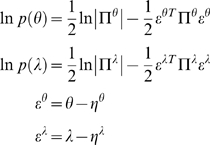
(14)


#### Summary

In this section, we have introduced hierarchical dynamic models in generalised coordinates of motion. These models are about as complicated as one could imagine; they comprise causes and hidden states, whose dynamics can be coupled with arbitrary (analytic) nonlinear functions. Furthermore, these states can have random fluctuations with unknown amplitude and arbitrary (analytic) autocorrelation functions. A key aspect of the model is its hierarchical form, which induces empirical priors on the causes. These recapitulate the constraints on hidden states, furnished by the hierarchy implicit in generalised motion. We now consider how these models are inverted.

### Model Inversion

This section considers variational inversion of models under mean-field and Laplace approximations, with a special focus on HDMs. This treatment provides a heuristic summary of the material in [2]. Variational Bayes is a generic approach to model inversion that approximates the conditional density *p*(*ϑ*|*y*,*m*) on some model parameters, *ϑ*, given a model *m* and data *y*. This is achieved by optimising the sufficient statistics (e.g., mean and variance) of an approximate conditional density *q*(*ϑ*)with respect to a lower bound on the evidence (marginal or integrated likelihood) *p*(*y*|*m*) of the model itself. These two quantities are used for inference on the parameters of any given model and on the model *per se*. [11]–[15]. The log-evidence for any parametric model can be expressed in terms of a free-energy *F*(*ỹ*,*q*) and a divergence term, for any density *q*(*ϑ*) on the unknown quantities
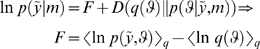
(15)The free-energy comprises the internal energy, *U*(*y*,*ϑ*) = ln *p*(*y*,*ϑ*) expected under *q*(*ϑ*) and an entropy term, which is a measure of its uncertainty. In this paper, energies are the negative of the corresponding quantities in physics; this ensures the free-energy increases with log-evidence. Equation 15 indicates that *F*(*ỹ*,*q*) is a lower-bound on the log-evidence because the cross-entropy or divergence term is always positive.

The objective is to optimise *q*(*ϑ*) by maximising the free-energy and then use *F*≈ln *p*(*ỹ*|*m*) as a lower-bound approximation to the log-evidence for model comparison or averaging. Maximising the free-energy minimises the divergence, rendering *q*(*ϑ*)≈*p*(*ϑ*|*y*,*m*) an approximate posterior, which is exact for simple (e.g., linear) systems. This can then be used for inference on the parameters of the model selected.

Invoking an arbitrary density, *q*(*ϑ*) converts a difficult integration problem (inherent in computing the evidence; see discussion) into an easier optimisation problem. This rests on inducing a bound that can be optimised with respect to *q*(*ϑ*). To finesse optimisation, one usually assumes *q*(*ϑ*) factorises over a partition of the parameters
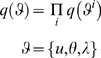
(16)In statistical physics this is called a mean-field approximation. This factorisation means that one assumes the dependencies between different sorts of parameters can be ignored. It is a ubiquitous assumption in statistics and machine learning. Perhaps the most common example is a partition into parameters coupling causes to responses and hyperparameters controlling the amplitude or variance of random effects. This partition greatly simplifies the calculation of things like *t*-tests and implies that, having seen some data, knowing their variance does not tell you anything more about their mean. Under our hierarchical dynamic model we will appeal to separation of temporal scales and assume, *q*(*ϑ*) = *q*(*u*(*t*))*q*(*θ*)*q*(*λ*), where *u* = [*ṽ*,*x̃*,]*^T^* are generalised states. This means that, in addition to the partition into parameters and hyperparameters, we assume conditional independence between quantities that change (states) and quantities that do not (parameters and hyperparameters).

In this dynamic setting *q*(*u*(*t*)) and the free-energy become functionals of time. By analogy with Lagrangian mechanics, this calls on the notion of *action*. Action is the anti-derivative or path-integral of energy. We will denote the action associated with the free energy by *F̅*, such that ∂*_t_F̅* = *F*. We now seek *q*(*ϑ^i^*) that maximise the action. It is fairly easy to show [2] that the solution for the states is a function of their instantaneous energy, *U*(*t*): = *U*(*u*|*θ*,*λ*) = ln *p*(*ỹ*,*u*|*θ*,*λ*)
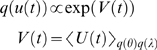
(17)where *V*(*t*) = ∂*_t_V̅* *^u^* is their variational energy. The variational energy of the states is simply their instantaneous energy averaged over their Markov blanket (i.e., averaged over the conditional density of the parameters and hyperparameters). Because the states are time-varying quantities, their conditional density is a function of time-dependent energy. In contrast, the conditional density of the parameters and hyperparameters are functions of their variational action, which are fixed for a given period of observation.
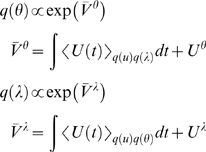
(18)Where *U^θ^* = ln *p*(*θ*) and *U^λ^* = ln *p*(*λ*) are the prior energies of the parameters and hyperparameters respectively and play the role of integration constants in the corresponding variational actions; *V̅* *^θ^* and *V̅* *^λ^*.

These equations provide closed-form expressions for the conditional or variational density in terms of the internal energy defined by our model; Equation 10. They are intuitively sensible, because the conditional density of the states should reflect the instantaneous energy; Equation 17. Whereas the conditional density of the parameters can only be determined after all the data have been observed; Equation 18. In other words, the variational energy involves the prior energy and an integral of time-dependent energy. In the absence of data, when the integrals are zero, the conditional density reduces to the prior density.

If the analytic forms of Equations 17 and 18 were tractable (e.g., through the use of conjugate priors), *q*(*ϑ^i^*) could be optimised directly by iterating these self-consistent nonlinear equations. This is known as variational Bayes; see [16] for an excellent treatment of static conjugate-exponential models. However, we will take a simpler approach that does not require bespoke update equations. This is based on a fixed-form approximation to the variational density.

#### The Laplace approximation

Under the Laplace approximation, the marginals of the conditional density assume a Gaussian form *q*(*ϑ^i^*) = *N*(*ϑ^i^*: *µ^i^*,*C^i^*) with sufficient statistics *µ^i^* and *C^i^*, corresponding to the conditional mean and covariance of the *i*th marginal. For consistency, we will use *µ^i^* for the conditional means or modes and *η^i^* for prior means. Similarly, we will use Σ*^i^* and *C^i^* for the prior and conditional covariances and Π*^i^* and *P^i^* for the corresponding inverses (i.e., precisions).

The advantage of the Laplace assumption is that the conditional covariance is a simple function of the modes. Under the Laplace assumption, the internal and variational actions are (ignoring constants)
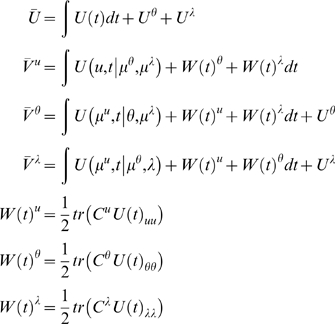
(19)
*C^u^* : = *C*(*t*)*^u^* is the conditional covariance of the states at time *t*∈[0,*N*]. The quantities *W*(*t*)*^i^* represent the contribution to the variational action from other marginals and mediate the effect of the uncertainty they encode on each other. We will refer to these as mean-field terms.

#### Conditional precisions

By differentiating Equation 19 with respect to the covariances and solving for zero, it is easy to show that the conditional precisions are the negative curvatures of the internal action [2]. Unless stated otherwise, all gradients and curvatures are evaluated at the mode or mean.
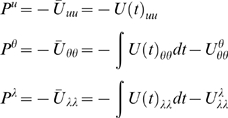
(20)Notice that the precisions of the parameters and hyperparameters increase with observation time, as one would expect. For our HDM the gradients and curvatures of the internal energy are
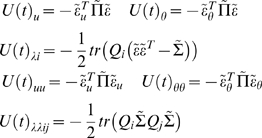
(21)where the covariance, 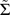 is the inverse of 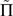. The *i*th element of the energy gradient; 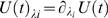 is the derivative with respect to the *i*th hyperparameter (similarly for the curvatures). We have assumed that the precision of the random fluctuations is linear in the hyperparameters, where 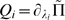, and 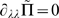. The derivatives of the generalised prediction error with respect to the generalised states are provided in Equation 12. The corresponding derivatives with respect to each parameter, 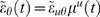 rest on second derivatives of the model's functions that mediate interactions between each parameter and the states
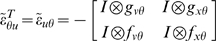
(22)These also quantify how the states and parameters affect each other through mean-field effects (see below).

#### Summary

The Laplace approximation gives a compact and simple form for the conditional precisions; and reduces the problem of inversion to finding the conditional modes. This generally proceeds in a series of iterated steps, in which the mode of each parameter set is updated. These updates optimise the variational actions in Equation 19 with respect to *µ^i^*, using the sufficient statistics (conditional mean and covariance) of the other sets. We have discussed static cases of this fixed-form scheme previously and how it reduces to expectation maximisation (**EM**; [17]) and restricted maximum likelihood (**ReML**; [18]) for linear models [15]. We now consider each of the steps entailed by our mean-field partition.

### Dynamic Expectation Maximisation

As with conventional variational schemes, we can update the modes of our three parameter sets in three distinct steps. However, the step dealing with the state (**D**-step) must integrate its conditional mode 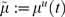 over time to accumulate the quantities necessary for updating the parameters (**E**-step) and hyperparameters (**M**-step). We now consider optimising the modes or conditional means in each of these steps.

#### The D-step

In static systems, the mode of the conditional density maximises variational energy, such that ∂*_u_V*(*t*) = 0; this is the solution to a gradient ascent scheme; 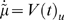. In dynamic systems, we also require the path of the mode to be the mode of the path; 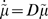. These two conditions are satisfied by the solution to the ansatz
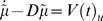
(23)Here 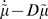 can be regarded as motion in a frame of reference that moves along the trajectory encoded in generalised coordinates. Critically, the stationary solution in this moving frame of reference maximises variational action. This can be seen easily by noting 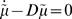 means the gradient of the variational energy is zero and
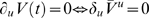
(24)This is sufficient for the mode to maximise variational action. In other words, changes in variational action, *V̅* *^u^*, with respect to variations of the path of the mode are zero (c.f., Hamilton's principle of stationary action). Intuitively, this means tiny perturbations to its path do not change the variational energy and it has the greatest variational action (i.e., path-integral of variational energy) of all possible paths.

Another way of looking at this is to consider the problem of finding the path of the conditional mode. However, the mode is in generalised coordinates and already encodes its path. This means we have to optimise the path of the mode subject to the constraint that 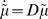, which ensures the path of the mode and the mode of the path are the same. The solution to Equation 23 ensures that variational energy is maximised and the path is self-consistent. Note that this is a very different (and simpler) construction in relation to incremental schemes such as Bayesian filtering.

Equation 23 prescribes the trajectory of the conditional mode, which can be realised with a local linearization [19] by integrating over Δ*t* to recover its evolution over discrete intervals
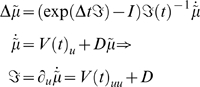
(25)For simplicity, we have suppressed the dependency of *V*(*u*,*t*) on the data. However, it is necessary to augment Equation 25 with any time-varying quantities that affect the variational energy. The form of the ensuing Jacobian ℑ(*t*) is
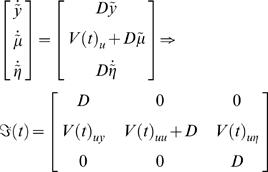
(26)Here. 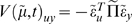 and 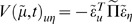 where
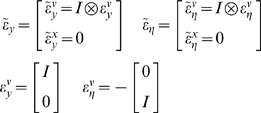
(27)These forms reflect the fact that data and priors only affect the prediction error at the first and last levels respectively. The only remaining quantities we require are the gradients and curvatures of the variational energy, which are simply
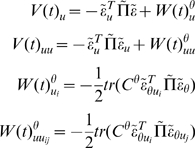
(28)The mean-field term, *W*(*t*)*^λ^* does not contribute to the **D**-step because it is not a function of the states. This means uncertainly about the hyperparameters does not affect the update for the states. This is because we assumed the precision was linear in the hyperparameters. The updates in Equation 25 provide the conditional trajectory 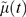 at each time point. Usually, Δ*t* is the time between observations but could be smaller, if nonlinearities in the model render local linearity assumptions untenable.

#### The E- and M-steps

Exactly the same update procedure can be used for the **E**- and **M**-steps. However, in this instance there are no generalised coordinates to consider. Furthermore, we can set the interval between updates to be arbitrarily long because the parameters are updated after the time-series has been integrated. If Δ*t*→∞ is sufficiently large, the matrix exponential in Equation 25 disappears (because the curvature of the Jacobian is negative definite) giving
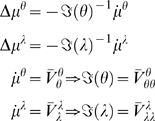
(29)Equation 29 is a conventional Gauss-Newton update scheme. In this sense, the **D**-Step can be regarded as a generalization of classical ascent schemes to generalised coordinates that cover dynamic systems. For our HDM, the requisite gradients and curvatures of variational action for the **E**-step are
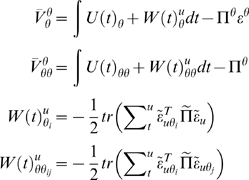
(30)Similarly, for the hyperparameters
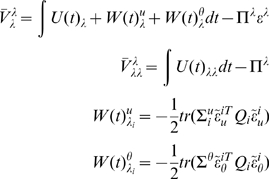
(31)Although uncertainty about the hyperparameters does not affect the states and parameters, uncertainty about both the states and parameters affect the hyperparameter update.

These steps represent a full variational scheme. A simplified version, which discounts uncertainty about the parameters and states in the **D** and **E**-steps, would be the analogue of an **EM** scheme. This simplification is easy to implement by removing *W*(*t*)*^θ^* and *W*(*t*)*^u^* from the **D-** and **E**-steps respectively. We will pursue this in the context of neurobiological implementations in the last section.

#### Summary

These updates furnish a variational scheme under the Laplace approximation. To further simplify things, we will assume Δ*t* = 1, such that sampling intervals serve as units of time. With these simplifications, the **DEM** scheme can be summarised as iterating until convergence

#### **D-**step **(states)**

*for t* = 1: *N*

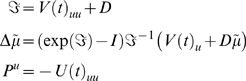



*end*

#### **E-**step **(parameters)**



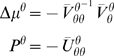



#### **M-**step **(hyperparameters)**



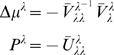
(32)


In this section, we have seen how the inversion of dynamic models can be formulated as an optimization of action. This action is the anti-derivative or path-integral of free-energy associated with changing states and a constant (of integration) corresponding to the prior energy of time-invariant parameters. By assuming a fixed-form (Laplace) approximation to the conditional density, one can reduce optimisation to finding the conditional modes of unknown quantities, because their conditional covariance is simply the curvature of the internal action (evaluated at the mode). The conditional modes of (mean-field) marginals optimise variational action, which can be framed in terms of gradient ascent. For the states, this entails finding a path or trajectory with stationary variational action. This can be formulated as a gradient ascent in a frame of reference that moves along the path encoded in generalised coordinates.

## Results

In this section, we review the model and inversion scheme of the previous section in light of established procedures for supervised and self-supervised learning. This section considers HDMs from the pragmatic point of view of statistics and machine learning, where the data are empirical and arrive as discrete data sequences. In the next section, we revisit these models and their inversion from the point of view of the brain, where the data are sensory and continuous. This section aims to establish the generality of HDMs by showing that many well-known approaches to data can be cast as an inverting a HDM under simplifying assumptions. It recapitulates the unifying perspective of Roweis and Ghahramani [20] with a special focus on hierarchical models and the triple estimation problems **DEM** can solve. We start with supervised learning and then move to unsupervised schemes. Supervised schemes are called for when causes are known but the parameters are not. Conversely, the parameters may be known and we may want to estimate causes or hidden states. This leads to a distinction between *identification* of a model's parameters and *estimation* of its states. When neither the states nor parameters are known, the learning is unsupervised. We will consider models in which the parameters are unknown, the states are unknown or both are unknown. Within each class, we will start with static models and then consider dynamic models.

All the schemes described in this paper are available in Matlab code as academic freeware (http://www.fil.ion.ucl.ac.uk/spm). The simulation figures in this paper can be reproduced from a graphical user interface called from the DEM toolbox.

### Models with Unknown Parameters

In these models the causes are known and enter as priors *η* with infinite precision; Σ*^v^* = 0. Furthermore, if the model is static or, more generally when *g_x_* = 0, we can ignore hidden states and dispense with the **D**-step.

#### Static models and neural networks

Usually, supervised learning entails learning the parameters of static nonlinear generative models with known causes. This corresponds to a HDM with infinitely precise priors at the last level, any number of subordinate levels (with no hidden states)
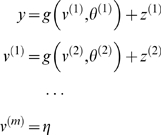
(33)One could regard this model as a neural network with *m* hidden layers. From the neural network perspective, the objective is to optimise the parameters of a nonlinear mapping from data *y* to the desired output *η*, using back propagation of errors or related approaches [21]. This mapping corresponds to inversion of the generative model that maps causes to data; *g* ^(*i*)^: *η*→*y*. This inverse problem is solved by **DEM**. However, unlike back propagation of errors or universal approximation in neural networks [22], **DEM** is not simply a nonlinear function approximation device. This is because the network connections parameterise a generative model as opposed to its inverse; *h*: *y*→*η* (i.e., recognition model). This means that the parameters specify how states cause data and can therefore be used to generate data. Furthermore, unlike many neural network or **PDP** (parallel distributed processing) schemes, **DEM** enables Bayesian inference through an explicit parameterisation of the conditional densities of the parameters.

#### Nonlinear system identification

In nonlinear optimisation, we want to identify the parameters of a static, nonlinear function that maps known causes to responses. This is a trivial case of the static model above that obtains when the hierarchical order reduces to *m* = 1
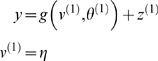
(34)The conditional estimates of *θ* ^(1)^ optimise the mapping *g* ^(1)^: *η*→*y* for any specified form of generating function. Because there are no dynamics, the generalised motion of the response is zero, rendering the **D**-step and generalised coordinates redundant. Therefore, identification or inversion of these models reduces to conventional expectation-maximisation (**EM**), in which the parameters and hyperparameters are optimised recursively, through a coordinate ascent on the variational energy implicit in the **E** and **M**-steps. Expectation-maximisation has itself some ubiquitous special cases, when applied to simple linear models:

#### The general linear model

Consider the linear model, with a response that has been elicited using known causes, *y* = *θ* ^(1)^ *η*+*z* ^(1)^. If we start with an initial estimate of the parameters, *θ* ^(1)^ = 0, the **E**-step reduces to
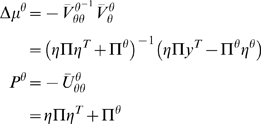
(35)These are the standard results for the conditional expectation and covariance of a general linear model, under parametric (i.e., Gaussian error) assumptions. From this perspective, the known causes *η^T^* play the role of explanatory variables that are referred to collectively in classical statistics as a design matrix. This can be seen more easily by considering the transpose of the linear model in Equation 34; *y^T^* = *η^T^θ* ^(1)*T*^+*z* ^(1)*T*^. In this form, the causes are referred to as explanatory or independent variables and the data as response or dependent variables. A significant association between these two sets of variables is usually established by testing the null hypothesis that *θ* ^(1)^ = 0. This proceeds either by comparing the evidence for (full or alternate) models with and (reduced or null) models without the appropriate explanatory variables or using the conditional density of the parameters, under the full model.

If we have flat priors on the parameters, Π*^θ^* = 0, the conditional moments in Equation (35) become maximum likelihood (**ML**) estimators. Finally, under i.i.d. (identically and independently distributed) assumptions about the errors, the dependency on the hyperparameters disappears (because the precisions cancel) and we obtain ordinary least squares (**OLS**) estimates; *µ^θ^* = *η* ^−^ *y^T^*, where *η* ^−^ = (*ηη^T^*)^−1^ *η* is the generalised inverse.

It is interesting to note that transposing the general linear model is equivalent to the switching the roles of the causes and parameters; *θ* ^(1)*T*^ ↔ *η*. Under this transposition, one could replace the **D**-step with the **E**-step. This gives exactly the same results because the two updates are formally identical for static models, under which
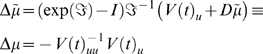
(36)The exponential term disappears because the update is integrated until convergence; i.e., Δ*t* = ∞. At this point, generalised motion is zero and an embedding order of *n* = 0⇒*D* = 0 is sufficient. This is a useful perspective because it suggests that static models can be regarded as models of steady-state or equilibrium responses, for systems with fixed point attractors.

#### Identifying dynamic systems

In the identification of nonlinear dynamic systems, one tries to characterise the architecture that transforms known inputs into measured outputs. This transformation is generally modelled as a generalised convolution [23]. When then inputs are known deterministic quantities the following *m* = 1 dynamic model applies
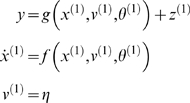
(37)Here *η* and *y* play the role of inputs (priors) and outputs (responses) respectively. Note that there is no state-noise; i.e., Σ*^w^* = 0 because the states are known. In this context, the hidden states become a deterministic nonlinear convolution of the causes [23]. This means there is no conditional uncertainty about the states (given the parameters) and the **D**-step reduces to integrating the state-equation to produce deterministic outputs. The **E**-Step updates the conditional parameters, based on the resulting prediction error and the **M**-Step estimates the precision of the observation error. The ensuing scheme is described in detail in [24], where it is applied to nonlinear hemodynamic models of fMRI time-series. This is an **EM** scheme that has been used widely to invert deterministic dynamic causal models of biological time-series. In part, the motivation to develop **DEM** was to generalise **EM** to handle state-noise or random fluctuations in hidden states. The extension of **EM** schemes into generalised coordinates had not yet been fully explored and represents a potentially interesting way of harnessing serial correlations in observation noise to optimise the estimates of a system's parameters. This extension is trivial to implement with **DEM** by specifying very high precisions on the causes and state-noise.

### Models with Unknown States

In these models, the parameters are known and enter as priors *η^θ^* with infinite precision, Σ*^θ^* = 0. This renders the **E**-Step redundant. We will review estimation under static models and then consider Bayesian deconvolution and filtering with dynamic models. Static models imply the generalised motion of causal states is zero and therefore it is sufficient to represent conditional uncertainty on their amplitude; i.e., *n* = 0⇒*D* = 0. As noted above the **D**-step for static models is integrated until convergence to a fixed point, which entails setting Δ*t* = ∞; see [15]. Note that making *n* = 0 renders the roughness parameter irrelevant because this only affects the precision of generalised motion.

#### Estimation with static models

In static systems, the problem reduces to estimating the causes of inputs after they are passed through some linear or nonlinear mapping to generate observed responses. For simple nonlinear estimation, in the absence of prior expectations about the causes, we have the nonlinear hierarchal model
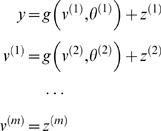
(38)This is the same as Equation 33 but with unknown causes. Here, the **D**-Step performs a nonlinear optimisation of the states to estimate their most likely values and the **M**-Step estimates the variance components at each level. As mentioned above, for static systems, Δ*t* = ∞ and *n* = 0. This renders it a classical Gauss-Newton scheme for nonlinear model estimation
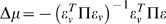
(39)Empirical priors are embedded in the scheme through the hierarchical construction of the prediction errors, *ε* and their precision Π, in the usual way; see Equation 11 and [15] for more details.

#### Linear models and parametric empirical Bayes

**Figure 3:**
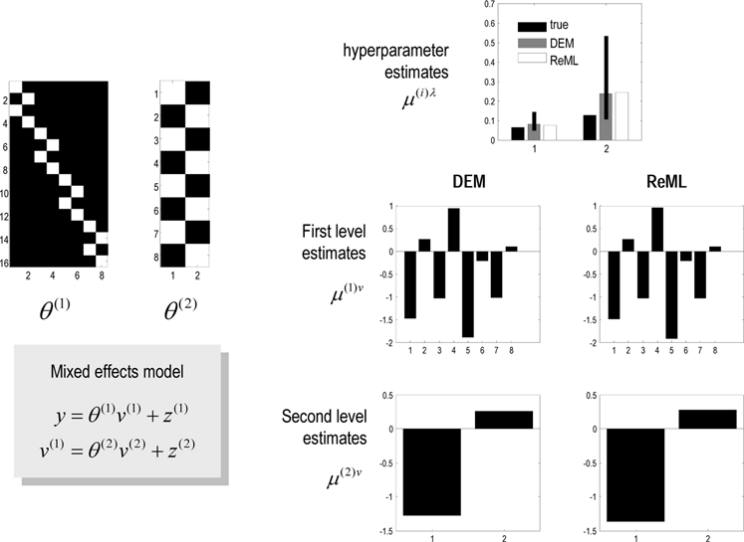
Example of estimation under a mixed-effects or hierarchical linear model. The inversion was cross-validated with expectation maximization (EM), where the M-step corresponds to restricted maximum likelihood (ReML). This example used a simple two-level model that embodies empirical shrinkage priors on the first-level parameters. These models are also known as parametric empirical Bayes (PEB) models (left). Causes were sampled from the unit normal density to generate a response, which was used to recover the causes, given the parameters. Slight differences in the hyperparameter estimates (upper right), due to a different hyperparameterisation, have little effect on the conditional means of the unknown causes (lower right), which are almost indistinguishable.

When the model above is linear, we have the ubiquitous hierarchical linear observation model used in Parametric Empirical Bayes (**PEB**; [8]) and mixed-effects analysis of covariance (**ANCOVA**) analyses.
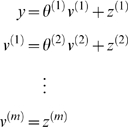
(40)Here the **D**-Step converges after a single iteration because the linearity of this model renders the Laplace assumption exact. In this context, the **M**-Step becomes a classical restricted maximum likelihood (**ReML**) estimation of the hierarchical covariance components, Σ^(*i*)*z*^. It is interesting to note that the **ReML** objective function and the variational energy are formally identical under this model [15],[18]. Figure 3 shows a comparative evaluation of **ReML** and **DEM** using the same data. The estimates are similar but not identical. This is because **DEM** hyperparameterises the covariance as a linear mixture of precisions, whereas the **ReML** scheme used a linear mixture of covariance components.

#### Covariance component estimation and Gaussian process models

When there are many more causes then observations, a common device is to eliminate the causes in Equation 40 by recursive substitution to give a model that generates sample covariances and is formulated in terms of covariance components (i.e., hyperparameters).
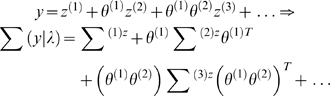
(41)Inversion then reduces to iterating the **M**-step. The causes can then be recovered from the hyperparameters using Equation 39 and the matrix inversion lemma. This can be useful when inverting ill-posed linear models (e.g., the electromagnetic inversion problem; [25]). Furthermore, by using shrinkage hyperpriors one gets a behaviour known as automatic relevance determination (**ARD**), where irrelevant components are essentially switched off [26]. This leads to sparse models of the data that are optimised automatically.

The model in Equation 41 is also referred to as a Gaussian process model [27]–[29]. The basic idea behind Gaussian process modelling is to replace priors *p*(*v*) on the parameters of the mapping, *g*(*v*): *v*→*y* with a prior on the space of mappings; *p*(*g*(*v*)). The simplest is a Gaussian process prior (**GPP**), specified by a Gaussian covariance function of the response, Σ(*y*|*λ*). The form of this **GPP** is furnished by the hierarchical structure of the HDM.

#### Deconvolution and dynamic models

In deconvolution problems, the objective is to estimate the inputs to a dynamic system given its response and parameters.
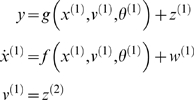
(42)This model is similar to Equation 37 but now we have random fluctuations on the unknown states. Estimation of the states proceeds in the **D**-Step. Recall the **E**-Step is redundant because the parameters are known. When Σ^(1)^ is known, the **M**-Step is also unnecessary and **DEM** reduces to deconvolution. This is related to Bayesian deconvolution or filtering under state-space models:

#### State-space models and filtering

State-space models have the following form in discrete time and rest on a vector autoregressive (**VAR**) formulation
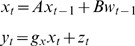
(43)where *w_t_* is a standard noise term. These models are parameterised by a system matrix *A*, an input matrix *B*, and an observation matrix *g_x_*. State-space models are special cases of linear HDMs, where the system-noise can be treated as a cause with random fluctuations
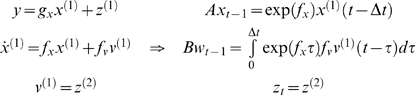
(44)Notice that we have had to suppress state-noise in the HDM to make a simple state-space model. These models are adopted by conventional approaches for inference on hidden states in dynamic models:

Deconvolution under HDMs is related to Bayesian approaches to inference on states using Bayesian belief update procedures (i.e., incremental or recursive Bayesian filters). The conventional approach to online Bayesian tracking of nonlinear or non-Gaussian systems employs extended Kalman filtering [30] or sequential Monte Carlo methods such as particle filtering. These Bayesian filters try to find the posterior densities of the hidden states in a recursive and computationally expedient fashion, assuming that the parameters and hyperparameters of the system are known. The extended Kalman filter is a generalisation of the Kalman filter in which the linear operators, of the state-space equations, are replaced by their partial derivatives evaluated at the current conditional mean. See also Wang and Titterington [31] for a careful analysis of variational Bayes for continuous linear dynamical systems and [32] for a review of the statistical literature on continuous nonlinear dynamical systems. These treatments belong to the standard class of schemes that assume Wiener or diffusion processes for state-noise and, unlike HDM, do not consider generalised motion.

In terms of establishing the generality of the HDM, it is sufficient to note that Bayesian filters simply estimate the conditional density on the hidden states of a HDM. As intimated in the introduction, their underlying state-space models assume that *z_t_* and *w_t_* are serially independent to induce a Markov property over sequential observations. This pragmatic but questionable assumption means the generalised motion of the random terms have zero precision and there is no point in representing generalised states. We have presented a fairly thorough comparative evaluation of **DEM** and extended Kalman filtering (and particle filtering) in [2]. **DEM** is consistently more accurate because it harvests empirical priors in generalised coordinates of motion. Furthermore, **DEM** can be used for both inference on hidden states and the random fluctuations driving them, because it uses an explicit conditional density *q*(*x̃*,*ṽ*) over both.

### Models with Unknown States and Parameters

In all the examples below, both the parameters and states are unknown. This entails a dual or triple estimation problem, depending on whether the hyperparameters are known. We will start with simple static models and work towards more complicated dynamic variants. See [33] for a comprehensive review of unsupervised learning for many of the models in this section. This class of models is often discussed under the rhetoric of blind source separation (BSS), because the inversion is blind to the parameters that control the mapping from sources or causes to observed signals.

#### Principal components analysis

The Principal Components Analysis (**PCA**) model assumes that uncorrelated causes are mixed linearly to form a static observation. This is a *m* = 1 model with no observation noise; i.e., Σ^(1)*z*^ = 0.
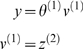
(45)where priors on *v* ^(1)^ = *z* ^(2)^ render them orthonormal Σ*^v^* = *I*. There is no **M**-Step here because there are no hyperparameters to estimate. The **D**-Step estimates the causes under the unitary shrinkage priors on their amplitude and the **E**-Step updates the parameters to account for the data. Clearly, there are more efficient ways of inverting this model than using **DEM**; for example, using the eigenvectors of the sample covariance of the data. However, our point is that **PCA** is a special case of an HDM and that any optimal solution will optimise variational action or energy. Nonlinear **PCA** is exactly the same but allowing for a nonlinear generating function.
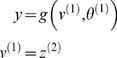
(46)See [34] for an example of nonlinear **PCA** with a bilinear model applied to neuroimaging data to disclose interactions among modes of brain activity.

#### Factor analysis and probabilistic PCA

**Figure 4:**
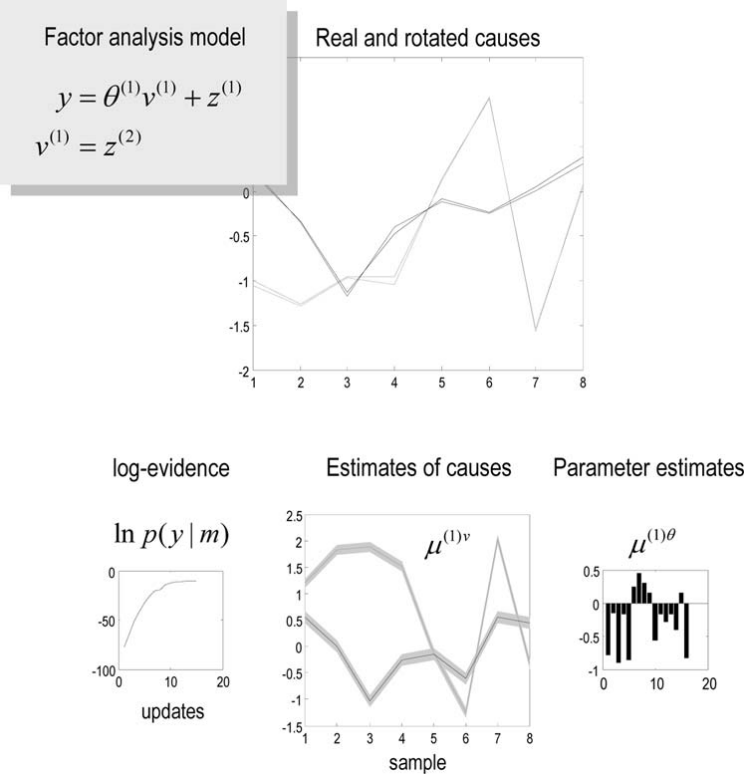
Example of Factor Analysis using a hierarchical model, in which the causes have deterministic and stochastic components. Parameters and causes were sampled from the unit normal density to generate a response, which was then used for their estimation. The aim was to recover the causes without knowing the parameters, which is effected with reasonable accuracy (upper). The conditional estimates of the causes and parameters are shown in lower panels, along with the increase in free-energy or log-evidence, with the number of DEM iterations (lower left). Note that there is an arbitrary affine mapping between the conditional means of the causes and their true values, which we estimated, *post hoc* to show the correspondence in the upper panel.

The model for factor analysis is exactly the same as for **PCA** but allowing for observation error
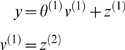
(47)When the covariance of the observation error is spherical; e.g., Σ^(1)*z*^ = *λ* ^(1)*z*^ *I*, this is also known as a probabilistic **PCA** model [35]. The critical distinction, from the point of view of the HDM, is that the **M**-Step is now required to estimate the error variance. See Figure 4 for a simple example of factor analysis using **DEM**. Nonlinear variants of factor analysis obtain by analogy with Equation 46.

#### Independent component analysis

Independent component analysis (**ICA**) decomposes the observed response into a linear mixture of non-Gaussian causes [36]. Non-Gaussian causal states are implemented simply in *m* = 2 hierarchical models with a nonlinear transformation at higher levels. **ICA** corresponds to
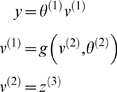
(48)Where, as for **PCA**, Σ*^v^* = *I*. The nonlinear function *g* ^(2)^ transforms a Gaussian cause, specified by the priors at the third level, into a non-Gaussian cause and plays the role of a probability integral transform. Note that there are no hyperparameters to estimate and consequently there is no **M**-Step. It is interesting to examine the relationship between nonlinear **PCA** and **ICA**; the key difference is that the nonlinearity is in the first level in **PCA**, as opposed to the second in **ICA**. Usually, in **ICA** the probability integral transform is pre-specified to render the second-level causes supra-Gaussian. From the point of view of a HDM this corresponds to specifying precise priors on the second-level parameters. However, **DEM** can fit unknown distributions by providing conditional estimates of both the mixing matrix *θ* ^(1)^ and the probability integral transform implicit in *g*(*v* ^(2)^,*θ* ^(2)^).

#### Sparse coding

In the same way that factor analysis is a generalisation of **PCA** to non-Gaussian causes, **ICA** can be extended to form sparse-coding models of the sort proposed by Olshausen and Fields [37] by allowing observation error.
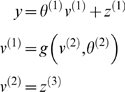
(49)This is exactly the same as the **ICA** model but with the addition of observation error. By choosing *g* ^(2)^ to create heavy-tailed (supra-Gaussian) second-level causes, sparse encoding is assured in the sense that the causes will have small values on most occasions and large values on only a few. Note the **M**-Step comes into play again for these models. All the models considered so far are for static data. We now turn to BSS in dynamic systems.

#### Blind deconvolution

Blind deconvolution tries to estimate the causes of an observed response without knowing the parameters of the dynamical system producing it. This represents the least constrained problem we consider and calls upon the same HDM used for system identification. An empirical example of triple estimation of states, parameters and hyperparameters can be found in [2]. This example uses functional magnetic resonance imaging time-series from a brain region to estimate not only the underlying neuronal and hemodynamic states causing signals but the parameters coupling experimental manipulations to neuronal activity. See Friston et al. [2] for further examples, ranging from the simple convolution model considered next, through to systems showing autonomous dynamics and deterministic chaos. Here we conclude with a simple *m* = 2 linear convolution model (Equation 42), as specified in Table 1.

**Table 1:** Specification of a linear convolution model.

Level	*g*(*x*,*v*)	*f*(*x*,*v*)	Π*^z^*	Π*^w^*	*η*(*t*)	*η^θ^*	Π*^θ^*	*η^λ^*	Π*^λ^*
*m* = 1	*θ*_1_ *x*	*θ*_2_ *x*+*θ*_3_ *v*	exp(*λ^z^*)	exp(*λ^w^*)		0	*e* ^−8^	0	*e* ^−16^
*m* = 2			1		0				

**Figure 5:**
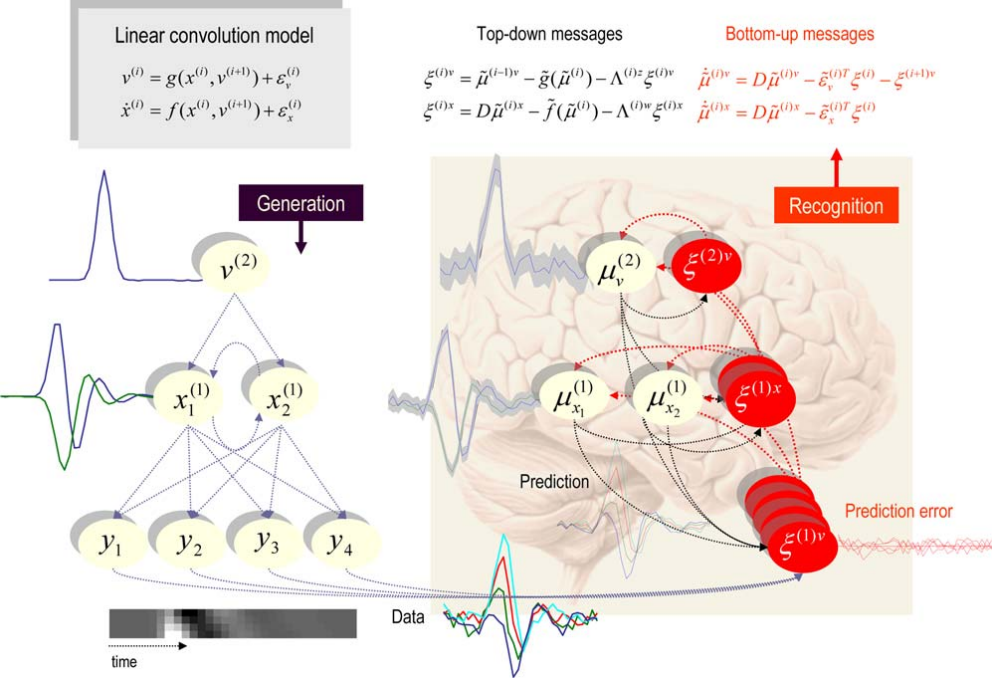
This schematic shows the linear convolution model used in the subsequent figure in terms of a directed Bayesian graph. In this model, a simple Gaussian ‘bump’ function acts as a cause to perturb two coupled hidden states. Their dynamics are then projected to four response variables, whose time-courses are cartooned on the left. This figure also summarises the architecture of the implicit inversion scheme (right), in which precision-weighted prediction errors drive the conditional modes to optimise variational action. Critically, the prediction errors propagate their effects up the hierarchy (c.f., Bayesian belief propagation or message passing), whereas the predictions are passed down the hierarchy. This sort of scheme can be implemented easily in neural networks (see last section and [5] for a neurobiological treatment). This generative model uses a single cause *v* ^(1)^, two dynamic states 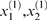 and four outputs *y*_1_,…,*y*_4_. The lines denote the dependencies of the variables on each other, summarised by the equations (in this example both the equations were simple linear mappings). This is effectively a linear convolution model, mapping one cause to four outputs, which form the inputs to the recognition model (solid arrow). The inputs to the four data or sensory channels are also shown as an image in the insert.

In this model, causes or inputs perturb the hidden states, which decay exponentially to produce an output that is a linear mixture of hidden states. Our example used a single input, two hidden states and four outputs. To generate data, we used a deterministic Gaussian bump function input *v* ^(1)^ = exp(^1^/_4_(*t*−12)^2^) and the following parameters
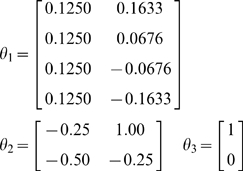
(50)During inversion, the cause is unknown and was subject to mildly informative (zero mean and unit precision) shrinkage priors. We also treated two of the parameters as unknown; one parameter from the observation function (the first) and one from the state equation (the second). These parameters had true values of 0.125 and −0.5, respectively, and uninformative shrinkage priors. The priors on the hyperparameters, sometimes referred to as hyperpriors were similarly uninformative. These Gaussian hyperpriors effectively place lognormal hyperpriors on the precisions (strictly speaking, this invalidates the assumption of a linear hyperparameterisation but the effects are numerically small), because the precisions scale as exp(*λ^z^*) and exp(*λ^w^*). Figure 5 shows a schematic of the generative model and the implicit recognition scheme based on prediction errors. This scheme can be regarded as a message passing scheme that is considered in more depth in the next section.

**Figure 6:**
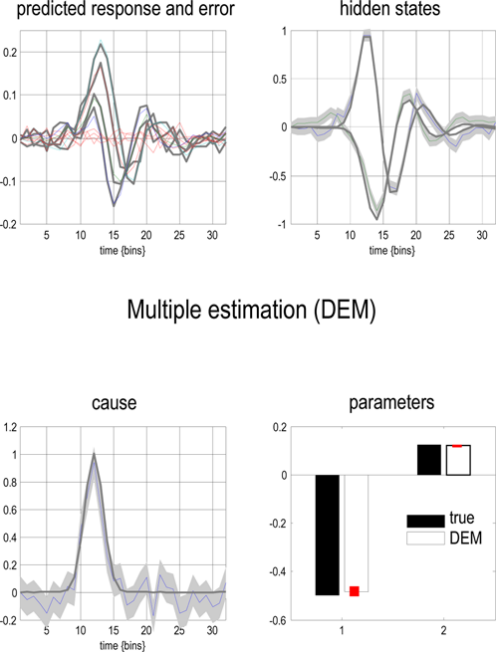
The predictions and conditional densities on the states and parameters of the linear convolution model of the previous figure. Each row corresponds to a level, with causes on the left and hidden states on the right. In this case, the model has just two levels. The first (upper left) panel shows the predicted response and the error on this response (their sum corresponds to the observed data). For the hidden states (upper right) and causes (lower left) the conditional mode is depicted by a coloured line and the 90% conditional confidence intervals by the grey area. These are sometimes referred to as “tubes”. Finally, the grey lines depict the true values used to generate the response. Here, we estimated the hyperparameters, parameters and the states. This is an example of triple estimation, where we are trying to infer the states of the system as well as the parameters governing its causal architecture. The hyperparameters correspond to the precision of random fluctuations in the response and the hidden states. The free parameters correspond to a single parameter from the state equation and one from the observer equation that govern the dynamics of the hidden states and response, respectively. It can be seen that the true value of the causal state lies within the 90% confidence interval and that we could infer with substantial confidence that the cause was non-zero, when it occurs. Similarly, the true parameter values lie within fairly tight confidence intervals (red bars in the lower right).

Figure 6 summarises the results after convergence of **DEM** (about sixteen iterations using an embedding order of *n* = 6, with a roughness hyperparameter, *γ* = 4). Each row corresponds to a level in the model, with causes on the left and hidden states on the right. The first (upper left) panel shows the predicted response and the error on this response. For the hidden states (upper right) and causes (lower left) the conditional mode is depicted by a coloured line and the 90% conditional confidence intervals by the grey area. It can be seen that there is a pleasing correspondence between the conditional mean and veridical states (grey lines). Furthermore, the true values lie largely within the 90% confidence intervals; similarly for the parameters. This example illustrates the recovery of states, parameters and hyperparameters from observed time-series, given just the form of a model.

#### Summary

**Figure 7:**
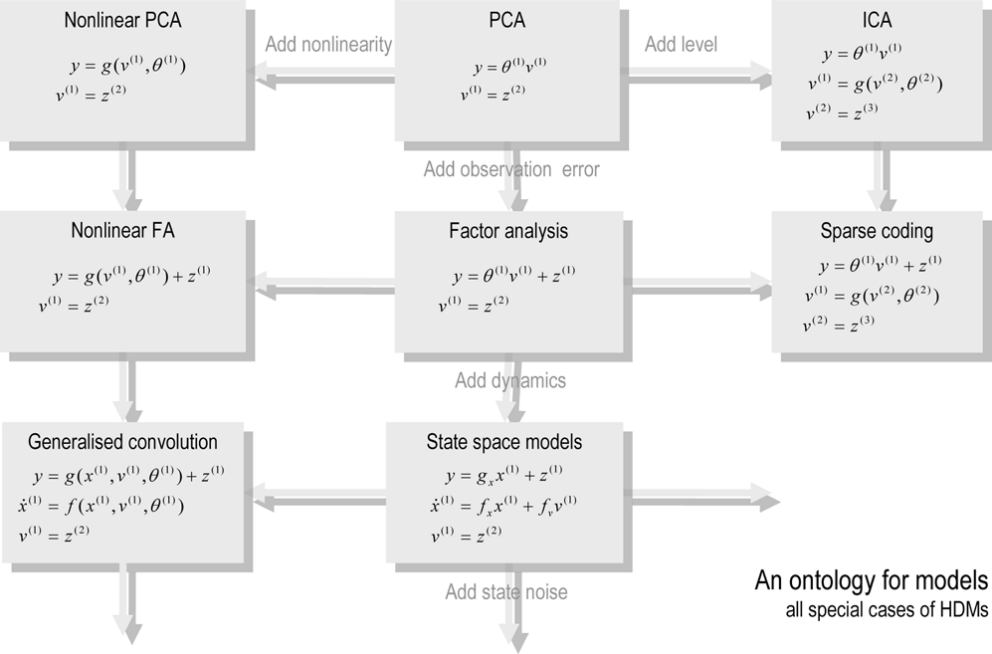
Ontology of models starting with a simple general linear model with two levels (the PCA model). This ontology is one of many that could be constructed and is based on the fact that hierarchical dynamic models have several attributes that can be combined to create an infinite number of models; some of which are shown in the figure. These attributes include; (i) the number of levels or depth; (ii) for each level, linear or nonlinear output functions; (iii) with or without random fluctuations; (iii) static or dynamic (iv), for dynamic levels, linear or nonlinear equations of motion; (v) with or without state noise and, finally, (vi) with or without generalised coordinates.

This section has tried to show that the HDM encompasses many standard static and dynamic observation models. It is further evident than many of these models could be extended easily within the hierarchical framework. Figure 7 illustrates this by providing a ontology of models that rests on the various constraints under which HDMs are specified. This partial list suggests that only a proportion of potential models have been covered in this section.

In summary, we have seen that endowing dynamical models with a hierarchical architecture provides a general framework that covers many models used for estimation, identification and unsupervised learning. A hierarchical structure, in conjunction with nonlinearities, can emulate non-Gaussian behaviours, even when random effects are Gaussian. In a dynamic context, the level at which the random effects enter controls whether the system is deterministic or stochastic and nonlinearities determine whether their effects are additive or multiplicative. **DEM** was devised to find the conditional moments of the unknown quantities in these nonlinear, hierarchical and dynamic models. As such it emulates procedures as diverse as independent components analysis and Bayesian filtering, using a single scheme. In the final section, we show that a **DEM**-like scheme might be implemented in the brain. If this is true, the brain could, in principle, employ any of the models considered in this section to make inferences about the sensory data it harvests.

### Neuronal Implementation

In this final section, we revisit **DEM** and show that it can be formulated as a relatively simple neuronal network that bears many similarities to real networks in the brain. We have made the analogy between the **DEM** and perception in previous communications; here we focus on the nature of recognition in generalised coordinates. In brief, deconvolution of hidden states and causes from sensory data (**D**-step) may correspond to perceptual inference; optimising the parameters of the model (**E**-step) may correspond to perceptual learning through changes in synaptic efficacy and optimising the precision hyperparameters (**M**-step) may correspond to encoding perceptual salience and uncertainty, through neuromodulatory mechanisms.

#### Hierarchical models in the brain

A key architectural principle of the brain is its hierarchical organisation [38]–[41]. This has been established most thoroughly in the visual system, where lower (primary) areas receive sensory input and higher areas adopt a multimodal or associational role. The neurobiological notion of a hierarchy rests upon the distinction between forward and backward connections [42]–[45]. This distinction is based upon the specificity of cortical layers that are the predominant sources and origins of extrinsic connections (extrinsic connections couple remote cortical regions, whereas intrinsic connections are confined to the cortical sheet). Forward connections arise largely in superficial pyramidal cells, in supra-granular layers and terminate on spiny stellate cells of layer four in higher cortical areas [40],[46]. Conversely, backward connections arise largely from deep pyramidal cells in infra-granular layers and target cells in the infra and supra-granular layers of lower cortical areas. Intrinsic connections mediate lateral interactions between neurons that are a few millimetres away. There is a key functional asymmetry between forward and backward connections that renders backward connections more modulatory or nonlinear in their effects on neuronal responses (e.g., [44]; see also Hupe et al. [47]). This is consistent with the deployment of voltage-sensitive NMDA receptors in the supra-granular layers that are targeted by backward connections [48]. Typically, the synaptic dynamics of backward connections have slower time constants. This has led to the notion that forward connections are driving and illicit an obligatory response in higher levels, whereas backward connections have both driving and modulatory effects and operate over larger spatial and temporal scales.

The hierarchical structure of the brain speaks to hierarchical models of sensory input. We now consider how this functional architecture can be understood under the inversion of HDMs by the brain. We first consider inference on states or perception.

#### Perceptual inference

If we assume that the activity of neurons encode the conditional mode of states, then the **D**-step specifies the neuronal dynamics entailed by perception or recognizing states of the world from sensory data. Furthermore, if we ignore mean-field terms; i.e., discount the effects of conditional uncertainty about the parameters when optimising the states, Equation 23 prescribes very simple recognition dynamics
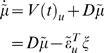
(51)Where, 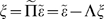 is prediction error multiplied by its precision, which we have re-parameterised in terms of a covariance component, 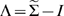. Here, the matrix Λ can be thought of as lateral connections among error-units. Equation 51 is an ordinary differential equation that describes how neuronal states self-organise, when exposed to sensory input. The form of Equation 51 is quite revealing, it suggests two distinct populations of neurons; *state-units* whose activity encodes 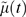 and *error-units* encoding *ξ*(*t*), with one error-unit for each state. Furthermore, the activities of error-units are a function of the states and the dynamics of state-units are a function of prediction error. This means the two populations pass messages to each other and to themselves. The messages passed among the states, 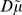 mediate empirical priors on their motion, while the lateral connections among the error-units, −Λ*ξ* weight prediction errors in proportion to their precision.

#### Hierarchical message passing

**Figure 8:**
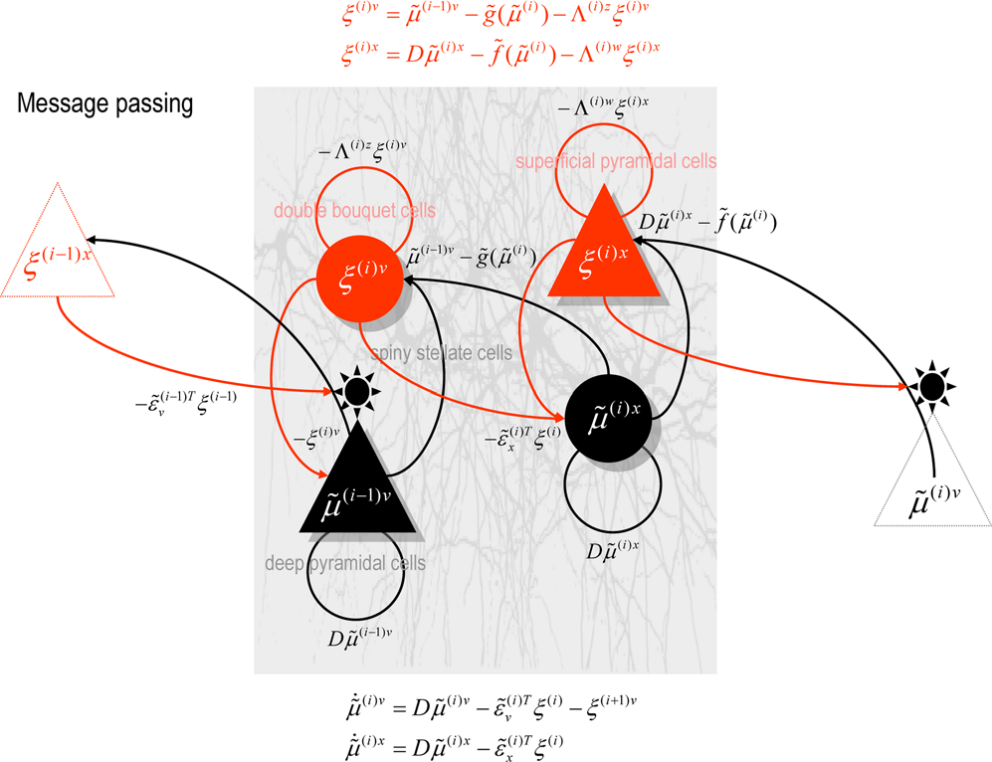
Schematic detailing the neuronal architectures that encode an ensemble density on the states and parameters of one level in a hierarchical model. This schematic shows the speculative cells of origin of forward driving connections that convey prediction error from a lower area to a higher area and the backward connections that are used to construct predictions. These predictions try to explain away input from lower areas by suppressing prediction error. In this scheme, the sources of forward connections are the superficial pyramidal cell population and the sources of backward connections are the deep pyramidal cell population. The differential equations relate to the optimisation scheme detailed in the main text and their constituent terms are placed alongside the corresponding connections. The state-units and their efferents are in black and the error-units in red, with causes on the left and hidden states on the right. For simplicity, we have assumed the output of each level is a function of, and only of, the hidden states. This induces a hierarchy over levels and, within each level, a hierarchical relationship between states, where hidden states predict causes.

If we unpack these equations we can see the hierarchical nature of this message passing (see Figure 8).
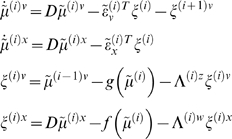
(52)This shows that error-units receive messages from the states in the same level and the level above, whereas states are driven by error-units in the same level and the level below. Critically, inference requires only the prediction error from the lower level *ξ* ^(*i*)^ and the level in question, *ξ* ^(*i*+1)^. These constitute bottom-up and lateral messages that drive conditional means 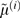 towards a better prediction, to explain away the prediction error in the level below. These top-down and lateral predictions correspond to *g̃* ^(*i*)^ and *f̃* ^(*i*)^. This is the essence of recurrent message passing between hierarchical levels to optimise free-energy or suppress prediction error; i.e., recognition dynamics.

The connections from error to state-units have a simple form that depends on the gradients of the model's functions; from Equation 12
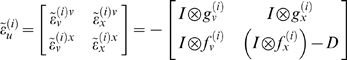
(53)These pass prediction errors forward to state-units in the higher level and laterally to state-units at the same level. The reciprocal influences of the state on the error-units are mediated by backward connections and lateral interactions. In summary, all connections between error and state-units are reciprocal, where the only connections that link levels are forward connections conveying prediction error to state-units and reciprocal backward connections that mediate predictions (see Figure 8).

**Figure 9:**
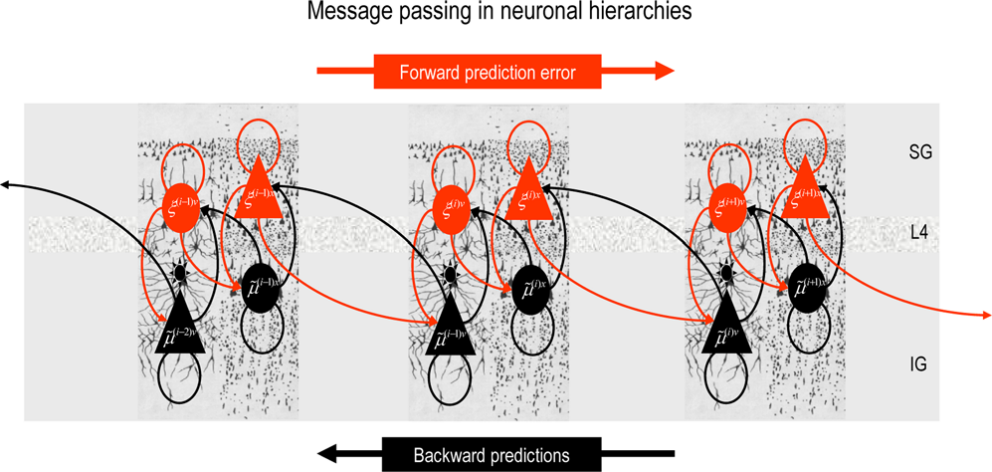
Schematic detailing the neuronal architectures that encode an ensemble density on the states and parameters of hierarchical models. This schematic shows how the neuronal populations of the previous figure may be deployed hierarchically within three cortical areas (or macro-columns). Within each area the cells are shown in relation to the laminar structure of the cortex that includes supra-granular (SG) granular (L4) and infra-granular (IG) layers.

We can identify error-units with superficial pyramidal cells, because the only messages that pass up the hierarchy are prediction errors and superficial pyramidal cells originate forward connections in the brain. This is useful because it is these cells that are primarily responsible for electroencephalographic (EEG) signals that can be measured non-invasively. Similarly the only messages that are passed down the hierarchy are the predictions from state-units that are necessary to form prediction errors in lower levels. The sources of extrinsic backward connections are largely the deep pyramidal cells and one might deduce that these encode the expected causes of sensory states (see [49] and Figure 9). Critically, the motion of each state-unit is a linear mixture of bottom-up prediction error; see Equation 52. This is exactly what is observed physiologically; in that bottom-up driving inputs elicit obligatory responses that do not depend on other bottom-up inputs. The prediction error itself is formed by predictions conveyed by backward and lateral connections. These influences embody the nonlinearities implicit in *g̃* ^(*i*)^ and *f̃* ^(*i*)^. Again, this is entirely consistent with the nonlinear or modulatory characteristics of backward connections.

#### Encoding generalised motion

Equation 51 is cast in terms of generalised states. This suggests that the brain has an explicit representation of generalised motion. In other words, there are separable neuronal codes for different orders of motion. This is perfectly consistent with empirical evidence for distinct populations of neurons encoding elemental visual features and their motion (e.g., motion-sensitive area V5; [39]). The analysis in this paper suggests that acceleration and higher-order motion are also encoded; each order providing constraints on a lower order, through 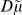. Here, *D* represents a fixed connectivity matrix that mediates these temporal constraints. Notice that 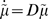 only when 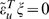. This means it is perfectly possible to represent the motion of a state that is inconsistent with the state of motion. The motion after-effect is a nice example of this, where a motion percept coexists with no change in the perceived location of visual stimuli. The encoding of generalised motion may mean that we represent paths or trajectories of sensory dynamics over short periods of time and that there is no perceptual instant (c.f., the remembered present; [50]). One could speculate that the encoding of different orders of motion may involve rate codes in distinct neuronal populations or multiplexed temporal codes in the same populations (e.g., in different frequency bands). See [51] for a neurobiologically realistic treatment of temporal dynamics in decision-making during motion perception and [52] for a discussion of synchrony and attentive learning in laminar thalamocortical circuits.

When dealing with empirical data-sequences one has to contend with sparse and discrete sampling. Analogue systems, like the brain can sample generalised motion directly. When sampling sensory data, one can imagine easily how receptors generate 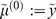. Indeed, it would be surprising to find any sensory system that did not respond to a high-order derivative of changing sensory fields (e.g., acoustic edge detection; offset units in the visual system, *etc*; [53]). Note that sampling high-order derivatives is formally equivalent to high-pass filtering sensory data. A simple consequence of encoding generalised motion is, in electrophysiological terms, the emergence of spatiotemporal receptive fields that belie selectivity to particular sensory trajectories.

#### Perceptual learning and plasticity

The conditional expectations of the parameters, *µ^θ^* control the construction of prediction error through backward and lateral connections. This suggests that they are encoded in the strength of extrinsic and intrinsic connections. If we define effective connectivity as the rate of change of a unit's response with respect to its inputs, Equation 51 suggests an interesting antisymmetry in the effective connectivity between the state and error-units. The effective connectivity from the states to the error-units is 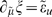. This is simply the negative transpose of the effective connectivity that mediates recognition dynamics; 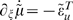. In other words, the effective connection from any state to any error-unit has the same strength (but opposite sign) of the reciprocal connection from the error to the state-unit. This means we would expect to see connections reciprocated in the brain, which is generally the case [39],[40]. Furthermore, we would not expect to see positive feedback loops; c.f., [54]. We now consider the synaptic efficacies underlying effective connectivity.

If synaptic efficacy encodes the parameter estimates, we can cast parameter optimisation as changing synaptic connections. These changes have a relatively simple form that is recognisable as associative plasticity. To show this, we will make the simplifying but plausible assumption that the brain's generative model is based on nonlinear functions *a* of linear mixtures of states
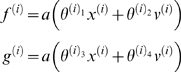
(54)Under this assumption 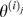 correspond to matrices of synaptic strengths or weights and *a* can be understood as a neuronal activation function that models nonlinear summation of presynaptic inputs over the dendritic tree [55]. This means that the synaptic connection to the *i*th error from the *j*th state depends on only one parameter, 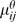 which changes according to Equation 29
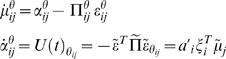
(55)This suggests that plasticity comprises an associative term 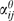 and a decay term mediating priors on the parameters. The dynamics of the associative term are given by Equation 21 (and exploiting the Kronecker form of Equation 22). The integral of this associative term is simply the covariance between presynaptic input and postsynaptic prediction error, summed over orders of motion. In short, it mediates associative or Hebbian plasticity. The product of pre and postsynaptic signals 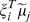 is modulated by an activity-dependent term, 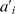, which is the gradient of the activation function at its current level of input (and is constant for linear models). Critically, updating the conditional estimates of the parameters, through synaptic efficacies, 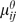, uses local information that is available at each error-unit. Furthermore, the same information is available at the synaptic terminal of the reciprocal connection, where the *i*th error-unit delivers presynaptic inputs to the *j*th state. In principle, this enables reciprocal connections to change in tandem. Finally, because plasticity is governed by two coupled ordinary differential equations (Equation 55), connection strengths should change more slowly than the neuronal activity they mediate. These theoretical predictions are entirely consistent with empirical and computational characterisations of plasticity [56],[57].

#### Perceptual salience and uncertainty

Equation 51 shows that the influence of prediction error is scaled by its precision 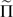 or covariance 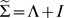 that is a function of *µ^λ^*. This means that the relative influence of bottom-up, lateral and top-down effects are modulated by the conditional expectation of the hyperparameters. This selective modulation of afferents mirrors the gain-control mechanisms invoked for attention; e.g., [58],[59]. Furthermore, they enact the sorts of mechanisms implicated in biased competition models of spatial and object-based attention mediating visual search [60],[61].

Equation 51 formulates this bias or gain-control in terms of lateral connections, 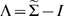 among error-units. This means hyperparameter optimisation would be realised, in the brain, as neuromodulation or plasticity of lateral interactions among error-units. If we assume that the covariance is a linear mixture of covariance components, *R_i_* among non-overlapping subsets of error-units, then
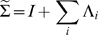
(56)Where 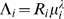. Under this hyperparameterisation, 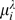 modulates subsets of connections to encode a partition of the covariance. Because each set of connections is a function of only one hyperparameter, their plasticity is prescribed simply by Equation 31
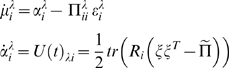
(57)The quantities 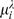 might correspond to specialised (e.g., noradrenergic or cholinergic) systems in the brain that broadcast their effects to the *i*th subset of error-units to modulate their responsiveness to each other. The activities of these units change relatively slowly, in proportion to an associative term 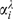 and decay that mediates hyperpriors. The associative term is basically the difference between the sample covariance of precision-weighted prediction errors and the precision expected, under the current value of 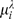.

As above, changes in 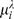 occur more slowly than the fast dynamics of the states because they are driven by 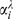, which accumulates energy gradients to optimise variational action. One could think of 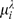 as the synaptic efficacy of lateral or intrinsic connections that depend upon classical neuromodulatory inputs and other slower synaptic dynamics (e.g., after-hyperpolarisation potentials and molecular signalling). The physiological aspects of these dynamics provide an interesting substrate for attentional mechanisms in the brain (see Schroeder et al., [62] for review) and are not unrelated to the ideas in [63]. These authors posit a role for acetylcholine (an ascending modulatory neurotransmitter) in mediating expected uncertainty. This is entirely consistent with the dynamics of 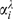 that are driven by the amplitude of prediction errors encoding the relative precision of sensory signals and empirical priors. Modulatory neurotransmitters have, characteristically, much slower time constants, in terms of their synaptic effects, than glutamatergic neurotransmission that is employed by cortico-cortical extrinsic connections.

#### The mean-field partition

**Figure 10:**
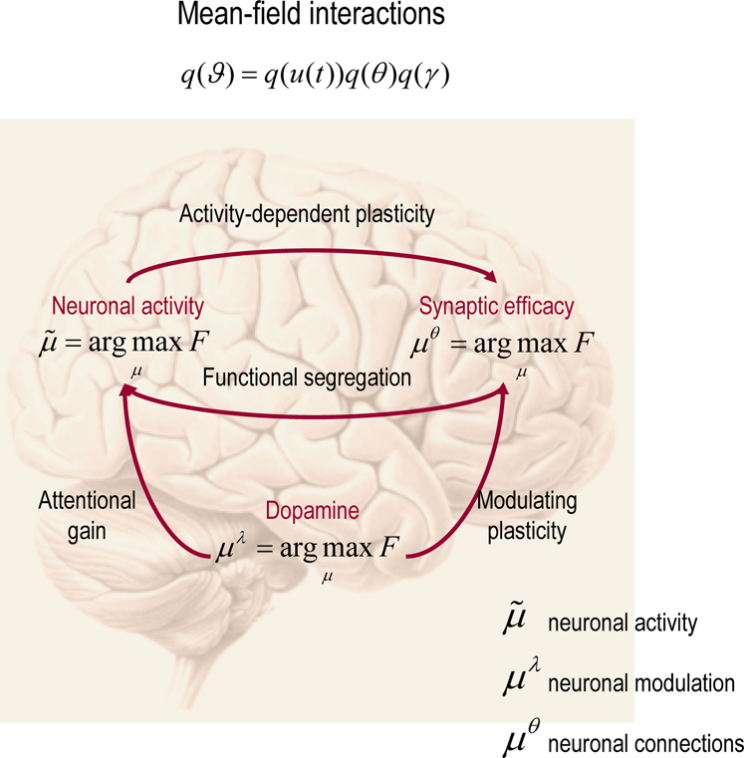
The ensemble density and its mean-field partition. *q*(*ϑ*) is the ensemble density and is encoded in terms of the sufficient statistics of its marginals. These statistics or variational parameters (e.g., mean or expectation) change to extremise free-energy to render the ensemble density an approximate conditional density on the causes of sensory input. The mean-field partition corresponds to a factorization over the sets comprising the partition. Here, we have used three sets (neural activity, modulation and connectivity). Critically, the optimisation of the parameters of any one set depends on the parameters of the other sets. In this figure, we have focused on means or expectations *µ^i^* of the marginal densities, *q*(*ϑ^i^*) = *N*(*ϑ^i^*: *µ^i^*,*C^i^*).

The mean-field approximation *q*(*ϑ*) = *q*(*u*(*t*))*q*(*θ*)*q*(*λ*) enables inference about perceptual states, causal regularities and context, without representing the joint distribution explicitly; c.f., [64]. However, the optimisation of one set of sufficient statistics is a function of the others. This has a fundamental implication for optimisation in the brain (see Figure 10). For example, ‘activity-dependent plasticity’ and ‘functional segregation’ speak to reciprocal influences between changes in states and connections; in that changes in connections depend upon activity and changes in activity depend upon connections. Things get more interesting when we consider three sets, because quantities encoding precision must be affected by and affect neuronal activity and plasticity. This places strong constraints on the neurobiological candidates for these hyperparameters. Happily, the ascending neuromodulatory neurotransmitter systems, such as dopaminergic and cholinergic projections, have exactly the right characteristics: they are driven by activity in presynaptic connections and can affect activity though classical neuromodulatory effects at the post-synaptic membrane [65], while also enabling potentiation of connection strengths [66],[67]. Furthermore, it is exactly these systems that have been implicated in value-learning [68]–[70], attention and the encoding of uncertainty [63],[71].

#### Summary

We have seen that the brain has, in principle, the infrastructure needed to invert hierarchical dynamic models of the sort considered in previous sections. It is perhaps remarkable that such a comprehensive treatment of generative models can be reduced to recognition dynamics that are as simple as Equation 51. Having said this, the notion that the brain inverts hierarchical models, using a **DEM**-like scheme, speaks to a range of empirical facts about the brain:

- The hierarchical organisation of cortical areas (c.f., [39])
- Each area comprises distinct neuronal subpopulations, encoding expected states of the world and prediction error (c.f., [72]).
- Extrinsic forward connections convey prediction error (from superficial pyramidal cells) and backward connections mediate predictions, based on hidden and causal states (from deep pyramidal cells) [49].
- Recurrent dynamics are intrinsically stable because they are trying to suppress prediction error [54],[64].
- Functional asymmetries in forwards (linear) and backwards (nonlinear) connections may reflect their distinct roles in recognition (c.f., [44]).
- Principal cells elaborating predictions (e.g., deep pyramidal cells) may show distinct (low-pass) dynamics, relative to those encoding error (e.g., superficial pyramidal cells)
- Lateral interactions may encode the relative precision of prediction errors and change in a way that is consistent with classical neuromodulation (c.f., [63],[71]).
- The rescaling of prediction errors by recurrent connections, in proportion to their precision, affords a form of cortical bias or gain control [73],[74].
- The dynamics of plasticity and modulation of lateral interactions encoding precision or uncertainty (which optimise a path-integral of variational energy) must be slower than the dynamics of neuronal activity (which optimise variational energy *per se*)
- Neuronal activity, synaptic efficacy and neuromodulation must all affect each other; activity-dependent plasticity and neuromodulation shape neuronal responses and:
- Neuromodulatory factors play a dual role in modulating postsynaptic responsiveness (e.g., through modulating in after-hyperpolarising currents) and synaptic plasticity [66],[67].

These observations pertain to the anatomy and physiology of neuronal architectures; see Friston et al. [5] for a discussion of operational and cognitive issues, under a free-energy principle for the brain.

We have tried to establish the generality of HDMs as a model that may be used by the brain. However, there are many alternative formulations that could be considered. Perhaps the work of Archambeau et al. [75] is formally the closest to one presented in this paper. These authors propose an approach that is very similar to DEM but is framed in terms of SDEs. A related formulation, with particle annihilation and symmetry breaking, has been proposed [76] as a mechanism for learning. This work adopts a path integral approach to optimal control theory and reinforcement learning. Cortical processing as the statistical result of the activity of neural ensembles is an established and important idea (e.g., [77],[78]). Although, computationally intensive (see Discussion), particle filtering can be efficient [79]; Furthermore, it can be combined with local linearised sequential methods (e.g., the Ensemble Kalman Filter; [80]) to provide data assimilation methods for huge data sets. In fact, Schiff and Sauer [81] have recently proposed an Ensemble Kalman Filter for the control of cortical dynamics that could have biological and engineering significance. Finally, [82] proposes a path integral approach to particle filtering for data assimilation. The common theme here is the use of ensembles to represent more realistic and complicated conditional densities. Although the biological relevance of these exciting developments remains to be established they may provide insights into neuronal computations. They also speak to ensembles of HDMs, under the Laplace assumption, to approximate the conditional density with a mixture of Gaussians (Nelson Trujillo-Barreto – personal communication).

Clearly, the theoretical treatment of this section calls for an enormous amount of empirical verification and hypothesis testing, not least to disambiguate among alternative theories and architectures. We have laid out the neurobiological and psychophysical motivation for the neuronal implementation of **DEM** in [3] and [4]. These papers deal with inference in the brain and motivate an overarching free-energy principle, using the notion of equilibrium densities and active agents. In [83] we address the face validity of the neuronal scheme described in this section, using synthetic birds and the perceptual categorisation of birdsong. These papers try to emulate empirical LFP and EEG studies to establish the sorts of electrophysiological responses one would expect to see in paradigms, such as those used to elicit the mismatch negativity [84],[85].

## Discussion

We are now in a position to revisit some of the basic choices behind the **DEM** scheme, in light of its neuronal implementation. Some of these choices are generic and some are specific to neuronal inversion. All can be framed in terms of assumptions about the existence and form of the approximating conditional density, *q*(*ϑ*). The first choice was to optimise a bound on the log-evidence, as opposed to the evidence itself. This choice is mandated by the fact that the evidence entails an integral
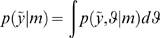
(58)that is not generally tractable. In other words, this integral has no general analytic solution, particularly when the generative model is nonlinear. This means the brain is obliged to induce a bound approximation, through *q*(*ϑ*).

The second choice was to use a fixed-form for *q*(*ϑ*), as opposed to a free-form. This is a more pragmatic choice that is dictated by implementational constraints. A fixed-form approximation allows one to represent the density in terms of a small number of quantities; its sufficient statistics. A free-form approximation would require an infinite number of quantities encoding the density over its support. Clearly, a fixed-form is assumption is imperative for the brain and predominates in most practical applications. In engineering and machine learning, free-form densities are usually approximated by the sample density of a large number of ‘particles’ that populate state-space. For dynamic systems in generalised coordinates, the ensuing scheme is known as variational filtering [1]. For standard SSMs, which ignore the high-order motion of random fluctuations, particle filtering is the most common approach. However, the dimensionality of the representational problems entailed by neuronal computations probably precludes particle-based (i.e., free-form) representations: consider face recognition, a paradigm example in perceptual inference. Faces can be represented in a perceptual space of about thirty dimensions (i.e., faces have about thirty discriminable attributes). To populate a thirty-dimensional space we would need at least 2^30^ particles, where each particle could correspond to the activity of thirty neurons (note that the conditional mean can be encoded with a single particle). The brain has about 2^11^ neurons at its disposal. Arguments like this suggest that free-form approximations and their attending sampling schemes are not really viable in a neuronal context (although they have been considered; see [86] and above)

The third choice was a mean-field approximation; *q*(*ϑ*) = *q*(*u*(*t*))*q*(*θ*)*q*(*λ*). This allowed us to separate the optimisation of states from parameters, using separation of temporal scales. This allowed us to optimise the states online, while learning the parameters offline. The motivation here was more didactic; in that special cases of the ensuing scheme are formally equivalent to established analyses of discrete data sequences (e.g., expectation maximisation and restricted maximum likelihood). However, the mean-field factorisation is less critical in neuronal implementations because the brain optimises both states and parameters online. We portrayed the neuronal implementation as a **DEM** scheme in which conditional uncertainty about the parameters was ignored when optimising the states and *vice versa* (i.e., mean field-effects were ignored). Alternatively, we could have relaxed the mean-field assumption and treated the solutions to Equations 51 and 52 as optimising the mean of *q*(*u*(*t*),*θ*) simultaneously. In this case, mean-field effects coupling states and parameters are no longer required.

The fourth assumption was that the fixed-form of *q*(*ϑ*) was Gaussian. This Laplace assumption affords an important simplification that may be relevant for recognition schemes that deal with large amounts of data. Under the Laplace assumption, only the conditional mean has to be optimised (because the conditional covariance is a function of the mean). The resulting recognition dynamics (Equation 51) are simple and neuronally plausible. The Laplace assumption enforces a unimodal approximation but does not require the density of underlying causes to be Gaussian. This is because nonlinearities in HDMs can implement probability integral transforms. A common example is the use of log-normal densities for non-negative scale parameters. These are simple to implement under the Laplace assumption with a log-transform, *ϑ* = ln *β*, which endows *β* with a log-normal density (we use this for precision hyperparameters; see the triple estimation example above). The unimodal constraint may seem restrictive; however, we know of no psychophysical or electrophysiological evidence for multimodal representations in the brain. In fact, the psychophysics of ambiguous stimuli and related bistable perceptual phenomena suggest that we can only represent one conditional cause or percept at a time.

The final choice was to include generalised motion under *q*(*ϑ*). The alternative would have been to assume the precisions of *z*′,*z*″,…*w*′,*w*″,…; the generalised motion of the random fluctuations, were zero (i.e., assume a serially uncorrelated process). It is important to appreciate that generalised motion always exists; the choice is whether to ignore it or not. Variational filtering and **DEM** assume high-order motion exists to infinite order. This is because random fluctuations in biophysical systems are almost invariably the product of dynamical systems, which renders their serial correlations analytic ([6], p 83; [23]). The resulting optimisation scheme is very simple (Equation 23) and is basically a restatement of Hamilton's principle of stationary action. If one ignores serial correlations, one could recourse to Extended Kalman filtering (EKF) or related Bayesian assimilation procedures for standard SSMs. From the perspective of **DEM**, these conventional procedures have an unduly complicated construction and deal only with a special (*n* = 0) case of dynamic models. In [2], we show that **DEM** and EKF give numerically identical results, when serial correlations are suppressed.

It is interesting to consider **DEM** in relation to common distinctions among inversion schemes: sequential data assimilation (SDA) vs. path integral approaches or integration *vs.* solution of differential equations. **DEM** blurs these distinctions somewhat: on the one hand, **DEM** is a path integral approach because the unknown quantities optimise action (the path integral of energy). On the other hand, it operates online and assimilates data with a differential equation (23), whose solution has stationary action. Furthermore, this equation can be integrated over time; indeed this is the mechanism suggested for neuronal schemes. However, when using **DEM** to analyse discrete data (e.g., the examples in the third section), this differential equation is solved over sampling intervals, using local linearization; c.f., [19].

### Summary

In summary, any generic inversion scheme needs to induce a lower-bound on the log-evidence by invoking an approximating conditional density *q*(*ϑ*) that, for dynamic systems, covers generalised motion. Physical constraints on the representation of *q*(*ϑ*) enforce a fixed parameterised form so that is can be encoded in terms of its parameters or sufficient statistics. The Laplace or Gaussian assumption about this fixed-form affords a substantial simplification of recognition dynamics at the price of restricting recognition to unimodal probabilistic representations; a price that evolution may well have paid to optimise neuronal schemes. The mean-field approximation is ubiquitous in the statistics but may not be necessary in an online or neuronal setting.

### Conclusion

In conclusion, we have seen how the inversion of a fairly generic hierarchical and dynamical model of sensory inputs can be transcribed onto neuronal quantities that optimise a variational bound on the evidence for that model This optimisation corresponds, under some simplifying assumptions, to suppression of prediction error at all levels in a cortical hierarchy. This suppression rests upon a balance between bottom-up (prediction error) influences and top-down (empirical prior) influences that are balanced by representations of their precision (uncertainty). These representations may be mediated by classical neuromodulatory effects and slow postsynaptic cellular processes that are driven by overall levels of prediction error.

The ideas presented in this paper have a long history, starting with the notion of neuronal energy [87]; covering ideas like efficient coding and analysis by synthesis [88],[89] to more recent formulations in terms of Bayesian inversion and predictive coding (e.g., [90],[91]). The specific contribution of this work is to establish the generality of models that may, at least in principle, be entertained by the brain.

## References

[1] Friston KJ (2008). Variational filtering.. Neuroimage.

[2] Friston KJ, Trujillo-Barreto N, Daunizeau J (2008). DEM: a variational treatment of dynamic systems.. Neuroimage.

[3] Friston KJ (2003). Learning and inference in the brain.. Neural Netw.

[4] Friston KJ (2005). A theory of cortical responses.. Philos Trans R Soc Lond B Biol Sci.

[5] Friston K, Kilner J, Harrison L (2006). A free energy principle for the brain.. J Physiol Paris.

[6] Stratonovich RL (1967). Topics in the Theory of Random Noise.

[7] Jazwinski AH (1970). Stochastic Processes and Filtering Theory.

[8] Kass RE, Steffey D (1989). Approximate Bayesian inference in conditionally independent
hierarchical models (parametric empirical Bayes models).. J Am Stat Assoc.

[9] Efron B, Morris C (1973). Stein's estimation rule and its competitors –
an empirical Bayes approach.. J Am Stats Assoc.

[10] Cox DR, Miller HD (1965). The theory of stochastic processes..

[11] Feynman RP (1972). Statistical mechanics.

[12] Hinton GE, von Cramp D (1993). Keeping neural networks simple by minimising the description
length of weights..

[13] MacKay DJC (1995). Free-energy minimisation algorithm for decoding and
cryptoanalysis.. Electron Lett.

[14] Neal RM, Hinton GE, Jordan MI (1998). A view of the EM algorithm that justifies incremental sparse and
other variants.. Learning in Graphical Models..

[15] Friston K, Mattout J, Trujillo-Barreto N, Ashburner J, Penny W (2007). Variational Bayes and the Laplace approximation.. Neuroimage.

[16] Beal MJ, Ghahramani Z, Bernardo JM, Bayarri MJ, Berger JO, Dawid AP, Heckerman D, Smith AFM, West M (2003). The variational Bayesian EM algorithm for incomplete Data: with
application to scoring graphical model structures.. Bayesian Statistics, Chapter 7..

[17] Dempster AP, Laird NM, Rubin DB (1977). Maximum likelihood from incomplete data via the EM algorithm.. J R Stat Soc Ser B.

[18] Harville DA (1977). Maximum likelihood approaches to variance component estimation
and to related problems.. J Am Stat Assoc.

[19] Ozaki T (1992). A bridge between nonlinear time-series models and nonlinear
stochastic dynamical systems: A local linearization approach.. Stat Sin.

[20] Roweis S, Ghahramani Z (1999). A unifying review of linear Gaussian models.. Neural Comput.

[21] Rumelhart DE, Hinton GE, Williams RJ, Rumelhart DE, McClelland JL (1986). Learning internal representations by error propagations.. Parallel Distributed Processing: Explorations in the Microstructures of
Cognition..

[22] Chen T, Chen H (1995). Universal approximation to nonlinear operators by neural networks
with arbitrary activation functions and its application to dynamical
systems.. IEEE Trans Neural Netw.

[23] Fliess M, Lamnabhi M, Lamnabhi-Lagarrigue F (1983). An algebraic approach to nonlinear functional expansions.. IEEE Trans Circuits Syst.

[24] Friston KJ (2002). Bayesian estimation of dynamical systems: an application to fMRI.. Neuroimage.

[25] Mattout J, Phillips C, Penny WD, Rugg MD, Friston KJ (2006). MEG source localization under multiple constraints: an extended
Bayesian framework.. Neuroimage.

[26] Tipping ME (2001). Sparse Bayesian learning and the Relevance Vector Machine.. J Mach Learn Res.

[27] Ripley BD, Cherkassy V, Friedman JH, Wechsler H (1994). Flexible Nonlinear Approaches to Classification.. From Statistics to Neural Networks..

[28] Rasmussen CE (1996). Evaluation of Gaussian Processes and Other Methods for Nonlinear
Regression [PhD thesis]. Toronto, Canada: Department of
Computer Science, University of Toronto.. http://www.cs.utoronto.ca/~carl.

[29] Kim H-C, Ghahramani Z (2006). Bayesian Gaussian process classification with the EM-EP
algorithm.. IEEE Trans Pattern Anal Mach Intell.

[30] Kalman R (1960). A new approach to linear filtering and prediction problems.. ASME Trans J Basic Eng.

[31] Wang B, Titterington DM (2004). Variational Bayesian inference for partially observed diffusions.
Technical Report 04-4, University of Glasgow.. http://www.stats.gla.ac.uk/Research/-TechRep2003/04-4.pdf.

[32] Sørensen H (2004). Parametric inference for diffusion processes observed at discrete
points in time: a survey.. Int Stat Rev.

[33] Ghahramani Z, Bousquet O, Raetsch G, von Luxburg U (2004). Unsupervised Learning.. Advanced Lectures on Machine Learning LNAI 3176..

[34] Friston K, Phillips J, Chawla D, Büchel C (2000). Nonlinear PCA: characterizing interactions between modes of brain
activity.. Philos Trans R Soc Lond B Biol Sci.

[35] Tipping ME, Bishop C (1999). Probabilistic principal component analysis.. J R Stat Soc Ser B.

[36] Bell AJ, Sejnowski TJ (1995). An information maximisation approach to blind separation and
blind de-convolution.. Neural Comput.

[37] Olshausen BA, Field DJ (1996). Emergence of simple-cell receptive field properties by learning a
sparse code for natural images.. Nature.

[38] Maunsell JH, van Essen DC (1983). The connections of the middle temporal visual area (MT) and their
relationship to a cortical hierarchy in the macaque monkey.. J Neurosci.

[39] Zeki S, Shipp S (1988). The functional logic of cortical connections.. Nature.

[40] Felleman DJ, Van Essen DC (1991). Distributed hierarchical processing in the primate cerebral
cortex.. Cereb Cortex.

[41] Mesulam MM (1998). From sensation to cognition.. Brain.

[42] Rockland KS, Pandya DN (1979). Laminar origins and terminations of cortical connections of the
occipital lobe in the rhesus monkey.. Brain Res.

[43] Murphy PC, Sillito AM (1987). Corticofugal feedback influences the generation of length tuning
in the visual pathway.. Nature.

[44] Sherman SM, Guillery RW (1998). On the actions that one nerve cell can have on another:
distinguishing “drivers” from
“modulators”.. Proc Natl Acad Sci U S A.

[45] Angelucci A, Levitt JB, Walton EJ, Hupe JM, Bullier J, Lund JS (2002). Circuits for local and global signal integration in primary
visual cortex.. J Neurosci.

[46] DeFelipe J, Alonso-Nanclares L, Arellano JI (2002). Microstructure of the neocortex: comparative aspects.. J Neurocytol.

[47] Hupe JM, James AC, Payne BR, Lomber SG, Girard P (1998). Cortical feedback improves discrimination between figure and
background by V1, V2 and V3 neurons.. Nature.

[48] Rosier AM, Arckens L, Orban GA, Vandesande F (1993). Laminar distribution of NMDA receptors in cat and monkey visual
cortex visualized by [3H]-MK-801 binding.. J Comp Neurol.

[49] Mumford D (1992). On the computational architecture of the neocortex. II. The role
of cortico-cortical loops.. Biol Cybern.

[50] Edelman GM (1993). Neural Darwinism: selection and reentrant signaling in higher
brain function.. Neuron.

[51] Grossberg S, Pilly P (2008). Temporal dynamics of decision-making during motion perception in
the visual cortex.. Vis Res.

[52] Grossberg S, Versace M (2008). Spikes, synchrony, and attentive learning by laminar
thalamocortical circuits.. Brain Res.

[53] Chait M, Poeppel D, de Cheveigné A, Simon JZ (2007). Processing asymmetry of transitions between order and disorder in
human auditory cortex.. J Neurosci.

[54] Crick F, Koch C (1998). Constraints on cortical and thalamic projections: the
no-strong-loops hypothesis.. Nature.

[55] London M, Häusser M (2005). Dendritic computation.. Annu Rev Neurosci.

[56] Buonomano DV, Merzenich MM (1998). Cortical plasticity: from synapses to maps.. Annu Rev Neurosci.

[57] Martin SJ, Grimwood PD, Morris RG (2000). Synaptic plasticity and memory: an evaluation of the hypothesis.. Annu Rev Neurosci.

[58] Treue S, Maunsell HR (1996). Attentional modulation of visual motion processing in cortical
areas MT and MST.. Nature.

[59] Martinez-Trujillo JC, Treue S (2004). Feature-based attention increases the selectivity of population
responses in primate visual cortex.. Curr Biol.

[60] Chelazzi L, Miller E, Duncan J, Desimone R (1993). A neural basis for visual search in inferior temporal cortex.. Nature.

[61] Desimone R (1996). Neural mechanisms for visual memory and their role in attention.. Proc Natl Acad Sci U S A.

[62] Schroeder CE, Mehta AD, Foxe JJ (2001). Determinants and mechanisms of attentional modulation of neural
processing.. Front Biosci.

[63] Yu AJ, Dayan P (2005). Uncertainty, neuromodulation and attention.. Neuron.

[64] Rao RP, Ballard DH (1998). Predictive coding in the visual cortex: a functional
interpretation of some extra-classical receptive field effects.. Nat Neurosci.

[65] Tseng KY, O'Donnell P (2004). Dopamine-glutamate interactions controlling prefrontal cortical
pyramidal cell excitability involve multiple signaling mechanisms.. J Neurosci.

[66] Brocher S, Artola A, Singer W (1992). Agonists of cholinergic and noradrenergic receptors facilitate
synergistically the induction of long-term potentiation in slices of rat
visual cortex.. Brain Res.

[67] Gu Q (2002). Neuromodulatory transmitter systems in the cortex and their role
in cortical plasticity.. Neuroscience.

[68] Friston KJ, Tononi G, Reeke GN, Sporns O, Edelman GM (1994). Value-dependent selection in the brain: simulation in a synthetic
neural model.. Neuroscience.

[69] Montague PR, Dayan P, Person C, Sejnowski TJ (1995). Bee foraging in uncertain environments using predictive Hebbian
learning.. Nature.

[70] Schultz W (2007). Multiple dopamine functions at different time courses.. Annu Rev Neurosci.

[71] Niv Y, Duff MO, Dayan P (2005). Dopamine, uncertainty and TD learning.. Behav Brain Funct.

[72] Kawato M, Hayakawa H, Inui T (1993). A forward-inverse optics model of reciprocal connections between
visual cortical areas.. Network.

[73] Desimone R, Duncan J (1995). Neural mechanisms of selective visual attention.. Annu Rev Neurosci.

[74] Abbott LF, Varela JA, Sen K, Nelson SB (1997). Synaptic depression and cortical gain control.. Science.

[75] Archambeau C, Cornford D, Opper M, Shawe-Taylor J (2007). Gaussian process approximations of stochastic differential
equations.. In: JMLR: Workshop and Conference Proceedings.

[76] Kappen HJ (2008). An introduction to stochastic control theory, path integrals and
reinforcement learning.. http://www.snn.ru.nl/~bertk/kappen_granada2006.pdf.

[77] John ER (1972). Switchboard versus statistical theories of learning and memory.. Science.

[78] Freeman WJ (2008). A pseudo-equilibrium thermodynamic model of information
processing in nonlinear brain dynamics.. Neural Netw.

[79] Beskos A, Papaspiliopoulos O, Roberts GO, Fearnhead P (2006). Exact and computationally efficient likelihood-based estimation
for discretely observed diffusion processes (with discussion).. J R Stat Soc Ser B.

[80] Evensen G, van Leeuwen PJ (2000). An ensemble Kalman smoother for nonlinear dynamics.. Mon Weather Rev.

[81] Schiff SJ, Sauer T (2008). Kalman filter control of a model of spatiotemporal cortical
dynamics.. J Neural Eng.

[82] Restrepo JM (2008). A path integral method for data assimilation.. Physica D.

[83] Friston KJ, Kiebel S (2009). Predictive coding under the free energy principle..

[84] Henson R, Shallice T, Dolan R (2000). Neuroimaging evidence for dissociable forms of repetition
priming.. Science.

[85] Näätänen R (2003). Mismatch negativity: clinical research and possible applications.. Int J Psychophysiol.

[86] Lee TS, Mumford D (2003). Hierarchical Bayesian inference in the visual cortex.. J Opt Soc Am A.

[87] Helmholtz H, Southall JPC (1860/1962). Handbuch der Physiologischen Optik. English translation..

[88] Barlow HB, Rosenblith WA (1961). Possible principles underlying the transformation of sensory
messages.. Sensory Communication..

[89] Neisser U (1967). Cognitive psychology.

[90] Ballard DH, Hinton GE, Sejnowski TJ (1983). Parallel visual computation.. Nature.

[91] Dayan P, Hinton GE, Neal RM (1995). The Helmholtz machine.. Neural Comput.

